# A novel hybrid protein composed of superoxide-dismutase-active Cu(II) complex and lysozyme

**DOI:** 10.1038/s41598-023-33926-1

**Published:** 2023-04-27

**Authors:** Tetsundo Furuya, Daisuke Nakane, Kenichi Kitanishi, Natsuki Katsuumi, Arshak Tsaturyan, Igor N. Shcherbakov, Masaki Unno, Takashiro Akitsu

**Affiliations:** 1 Department of Chemistry, Faculty of Science, Tokyo University of Science, 1-3 Kagurazaka, Shinjuku-ku, Tokyo, 162-8601 Japan; 2 Université Jean Monnet Saint-Etienne, CNRS, Institut d Optique Graduate School, Laboratoire Hubert Curien UMR 5516, 42023 Saint-Étienne, France; 3 Institute of Physical and Organic Chemistry, Southern Federal University, 194/2 Stachka Ave., Rostov-On-Don, 344090 Russia; 4 Department of Chemistry, Southern Federal University, 7 Zorge Str., Rostov-On-Don, 344090 Russia; 5 Graduate School of Science and Engineering, Ibaraki University, 4-12-1 Nakanarusawa, Hitachi, Ibaraki, 316-8511 Japan

**Keywords:** Biochemistry, Chemistry

## Abstract

A novel hybrid protein composed of a superoxide dismutase-active Cu(II) complex (CuST) and lysozyme (CuST@lysozyme) was prepared. The results of the spectroscopic and electrochemical analyses confirmed that CuST binds to lysozyme. We determined the crystal structure of CuST@lysozyme at 0.92 Å resolution, which revealed that the His15 imidazole group of lysozyme binds to the Cu(II) center of CuST in the equatorial position. In addition, CuST was fixed in position by the weak axial coordination of the Thr89 hydroxyl group and the hydrogen bond between the guanidinium group of the Arg14 residue and the hydroxyl group of CuST. Furthermore, the combination of CuST with lysozyme did not decrease the superoxide dismutase activity of CuST. Based on the spectral, electrochemical, structural studies, and quantum chemical calculations, an O_2_^–^ disproportionation mechanism catalyzed by CuST@lysozyme is proposed.

## Introduction

Aerobic organisms produce the energy necessary to sustain their lives through aerobic respiration. Reactive oxygen species (ROS) such as hydroxyl radicals (^·^OH), singlet oxygen (^1^O_2_), hydrogen peroxide (H_2_O_2_), and superoxide (O_2_^–^) are the inevitable byproducts of this process. These ROS cause serious oxidative damage to biomolecules such as lipids, carbohydrates, hormones, proteins, and nucleic acids. Among these ROS, O_2_^–^ is produced by electron transport systems, phagocytic processes, enzymatic oxidation, and oxygen-carrying proteins, such as hemoglobin and myoglobin^1^. Under protonic conditions, O_2_^–^ reacts with protons (H^+^) to produce other ROS, such as ^·^OH and H_2_O_2_^2^. Therefore, the removal of O_2_^–^ is a priority for aerobic organisms. To remove O_2_^–^ and avoid oxidative damage induced by O_2_^–^, aerobic organisms possess metalloenzymes known as superoxide dismutases (SODs). SODs catalyze the disproportionation of O_2_^–^ to H_2_O_2_ and O_2_, as shown in reaction (1):

1 $$2{\text{O}}_{2}^{ - } + \, 2{\text{H}}^{ + } \to {\text{H}}_{2} {\text{O}}_{2} + {\text{ O}}_{2}$$

Since SODs play a crucial role in protecting biomolecules from oxidative damage, the life expectancy of organisms relies on efficient SOD activity. Organisms with higher SOD activities have lower mortality rates and vice versa^3^. Metal ions lie in the active centers of SODs, which catalyze the disproportionation of O_2_^–^ to produce H_2_O_2_ and O_2_ via reactions (2) and (3), respectively:

2 $${\text{M}}^{{{\text{n}} + }} + {\text{ O}}_{2}^{ - } + 2{\text{H}}^{ + } \to {\text{M}}^{{\left( {{\text{n}} + 1} \right) + }} + {\text{ H}}_{2} {\text{O}}_{2}$$

3 $${\text{M}}^{{\left( {{\text{n}} + 1} \right) + }} + {\text{ O}}_{2}^{ - } \to {\text{M}}^{{{\text{n}} + }} + {\text{ O}}_{2}$$

Based on the metal ions present in the active centers, SODs are classified into four categories; SODs containing Ni, Fe, Mn, Cu, and Zn are known as NiSOD^4–8^, FeSOD^9–13^, MnSOD^14–19^, and CuZnSOD^20–22^, respectively. In this study, we focused on the most prevalent CuZnSOD, which contains Cu(II) and Zn(II) ions in its active center. While the Zn(II) ion fixes the secondary coordination structure around the active center^23^, the Cu(II) ion catalyzes the disproportionation reaction of O_2_^–^, as shown in reactions (4) and (5) below:

4 $${\text{Cu}}^{2 + } + {\text{ O}}_{2}^{ - } \to {\text{Cu}}^{ + } + {\text{ O}}_{2}$$

5 $${\text{Cu}}^{ + } + {\text{ O}}_{2}^{ - } + \, 2{\text{H}}^{ + } \to {\text{Cu}}^{2 + } + {\text{ H}}_{2} {\text{O}}_{2}$$

To use native CuZnSOD as an O_2_^–^ removal agent, issues such as its high cost and instability must be solved^24^. In this context, Cu(II) complexes with low molecular weights have been reported as functional SOD models^25,26^. Among these Cu(II) complexes, those coordinated by salicylic acid as a ligand have been reported as functional SOD models^26^. Our group also reported Cu(II) complexes composed of phenolate and L-amino acid moieties^27^. However, these coordination compounds can be toxic to biomolecules after the release of Cu(II) ions from their ligands^28^. To solve this problem, we focused on the strong Cu(II)-ion binding ability of proteins^29^.

In this study, as a first approach, we investigated the formation of a hybrid protein composed of lysozyme, which we chose owing to its stability and crystallinity, and a functional SOD-mimetic Cu(II) complex. This SOD-mimetic hybrid protein was expected to improve the biocompatibility and stability of the functional SOD model Cu(II) complex.

We also investigated and confirmed the formation of CuST-binding hybrid lysozyme (CuST@lysozyme) and elucidated its spectroscopic and electrochemical properties. Furthermore, we evaluated its SOD activity and suggested an O_2_^–^ disproportionation mechanism based on both experimental and computational studies.

## Results and discussion

### Strategy for hybrid protein formation

To form the hybrid protein, we focused on the imidazole group of only one His (His15) residue in lysozyme as the binding site for the Cu(II) complex. The presence of an imidazole group on the surface of lysozyme also aids the formation of a coordination bond with the metal ion. In fact, Mn(I)^30^, Ag(I)^31^, Au(I)^32^, and Pt(II)^33^ ions can bind to this lysozyme site. When the imidazole group of His15 functions as a binding site, the guanidinium groups of Arg14 and the hydroxyl group of Thr89 can interact with the Cu(II) complex through coordination bonds and/or hydrogen bonds.

**Figure 1:**
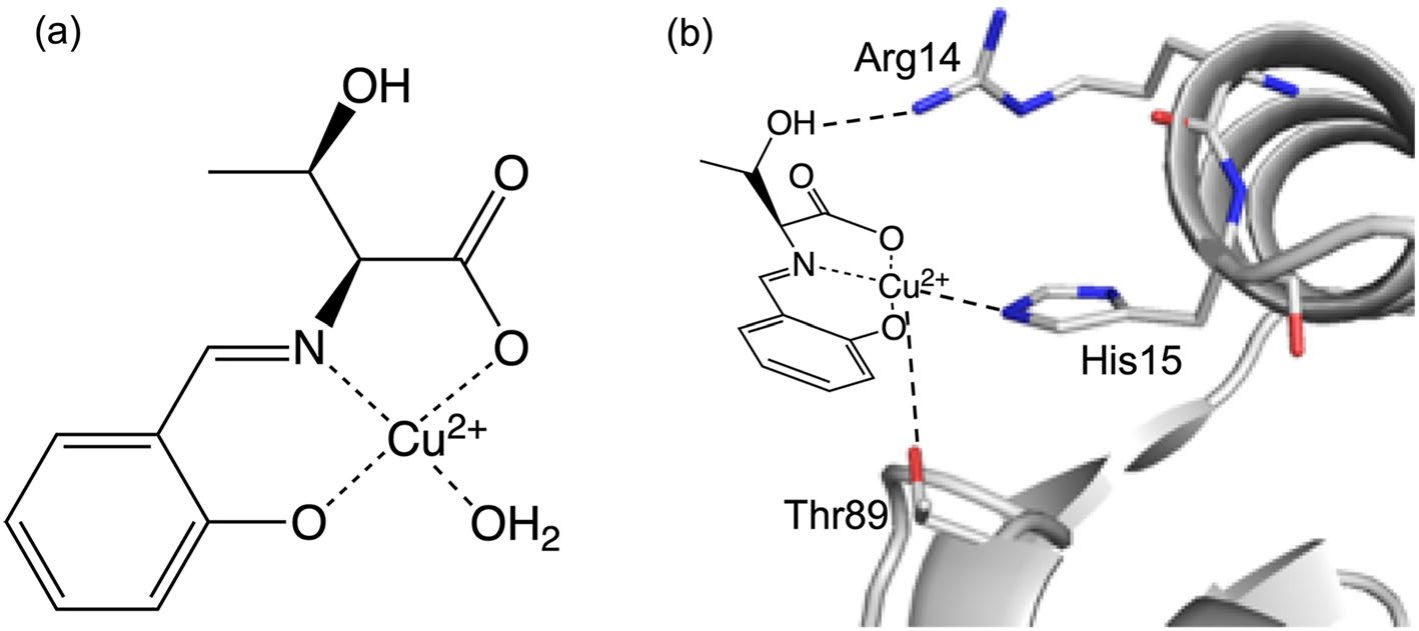
Schematic structures of (**a**) CuST, and (**b**) the estimated structure of the CuST-binding lysozyme.

In this context, we prepared a SOD functional model Cu(II) complex with a threonine derivative (CuST), as shown in Fig. 1a ^34^. A water molecule at the equatorial position of the Cu(II) complex is expected to be exchanged with the imidazole of the His15 residue to form the CuST-bound hybrid lysozyme. The proposed structure of the hybrid lysozyme is illustrated in Fig. 1b. Based on this structure, hydrogen bonds are expected to form between the hydroxyl group of CuST and the guanidinium group of the Arg14 side-chain, whereas the metal center of CuST is expected to be coordinated by the hydroxyl group of the Thr89 residue. These interactions between CuST and the functional groups on lysozyme can promote the rigid fixation of CuST.

### Preparation of CuST-Imi complex

In this study, we assumed that His15 binds to the Cu(II) center of CuST. To investigate the coordination behavior of imidazole, we prepared an imidazole-bound Cu(II) complex, CuST-Imi, by reacting CuST with an equivalent amount of imidazole.

### Crystal structure of CuST-Imi

Green, thin, plate-like, transparent CuST-Imi crystals suitable for X-ray crystallography were obtained by slow evaporation of the reaction mixture at room temperature (300 K) for 3–4 days.

**Figure 2:**
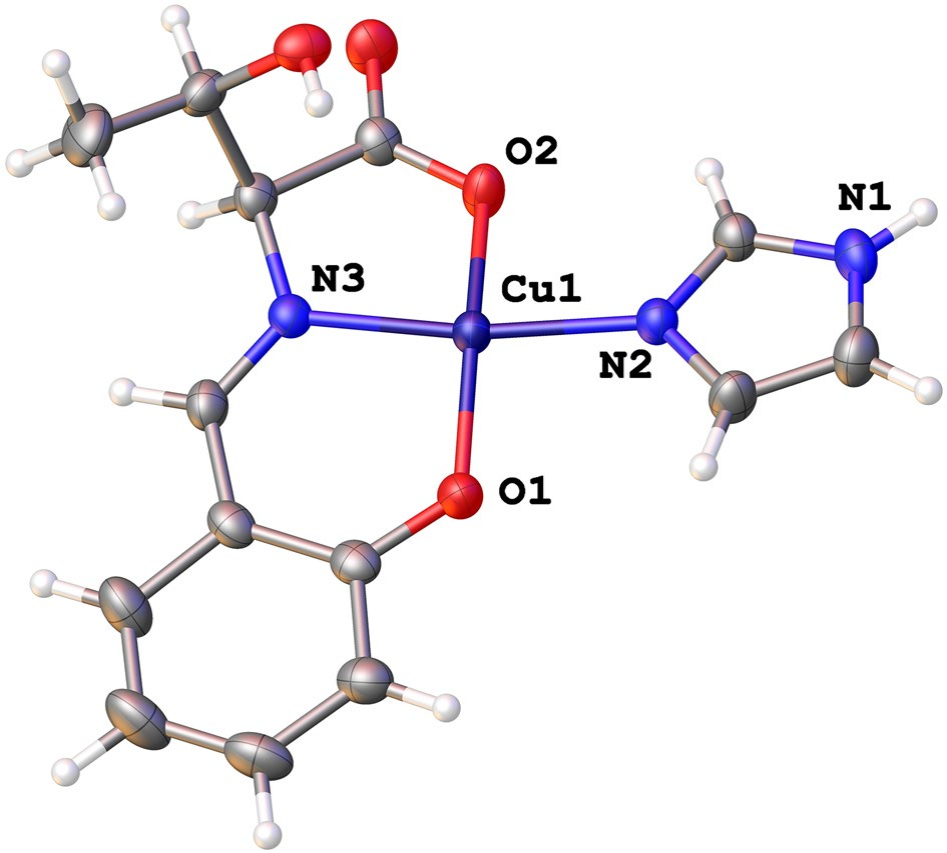
Crystal structure of CuST-Imi with thermal ellipsoids drawn at 50% probability. Selected bond lengths (Å) and angles (°): Cu1–O1 = 1.891(2), Cu1–O2 = 1.937(2), Cu1–N2 = 1.956(2), Cu1–N3 = 1.931(2), O1–Cu1–N3 = 95.21(9), N3–Cu1–O2 = 83.22(9), O1–Cu1–N2 = 92.87(8), O2–Cu1–N2 = 89.65(9), O1–Cu1–O2 = 165.30(8), and N2–Cu1–N3 = 171.46(9).

The crystallographic data collection and refinement statistics for CuST-Imi are summarized in Table S1. Single-crystal X-ray diffraction revealed that CuST-Imi crystallizes into the orthorhombic *P*2_1_2_1_2_1_ space group with *Z* = 4. As shown in Fig. 2, CuST-Imi exhibited a distorted square planar geometry. Furthermore, the Cu(II) center was bound to the phenolato O atom, imino N atom, and one of the carboxylate O atoms to form a tridentate structure; an additional imidazole ligand was bound to the remaining equatorial site of the Cu(II) center. The hydroxyl group of the Thr residue of the ligand did not coordinate with the Cu(II) center and was oriented to the outside of the core. The distance between the Cu(II) center and phenolato O atom (1.891 Å) was slightly shorter than the other Cu–N and Cu–O distances (1.931–1.956 Å). The shorter bond distance indicates that the Cu(II) ion, phenolato O atom, and imino N atom form a structurally stable six-membered chelate ring. Based on the N2–Cu–N3 (171.46°) and O1–Cu–O2 (165.30°) bond angles, the geometry of Cu(II) in CuST-Imi was slightly distorted from square planar.

### FT-IR spectroscopy

The FT-IR spectra of CuST and CuST-Imi are shown in Figure S1. Both CuST and CuST-Imi exhibited a C=N stretching vibration at 1633 cm^−1^. By contrast, the *ν*(COO^–^) asymmetric and *ν*(COO^–^) symmetric vibrations of the carboxyl group appeared in the ranges of 1542–1535 cm^–1^ and 1383–1382 cm^–1^, respectively. Based on the difference between the asymmetric and symmetric vibrations (≈ 200 cm^−1^), the carboxyl groups of CuST and CuST-Imi were concluded to bind to the Cu(II) centers in a unidentate manner^35^, and the FT-IR spectral features are consistent with the crystallographic analysis.

### UV–vis spectral properties of CuST and CuST-Imi

**Figure 3:**
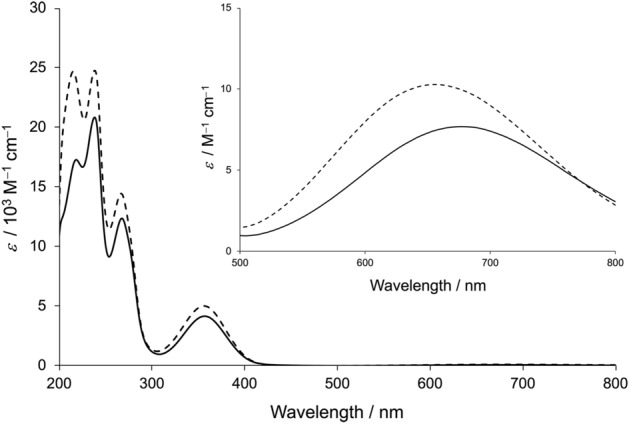
UV–vis spectra of CuST (0.1 mM, bold line) and CuST-Imi (0.1 mM, dashed line). Both spectra were measured in 0.1 M phosphate buffer (pH 7.0). Inset: expanded spectra in 500–800 nm region (1.0 mM).

The UV–vis spectra of CuST and CuST-Imi are shown in Fig. 3. CuST gave rise to a strong band at 356 nm and weak band at 678 nm, whereas CuST-Imi exhibited bands at 357 nm and 656 nm, respectively. Absorption bands in the UV region are attributed to ligand-to-metal charge transfer. On the other hand, the bands appearing in the lower-energy region are due to d–d transitions, indicating that these Cu(II) complexes have a compressed square-planar geometry^36^, consistent with the crystallographic analysis. Comparing their UV–vis spectra, the coordination of imidazole to the Cu(II) center of CuST gives a higher-energy d–d band in the visible region (678–656 nm).

**Figure 4:**
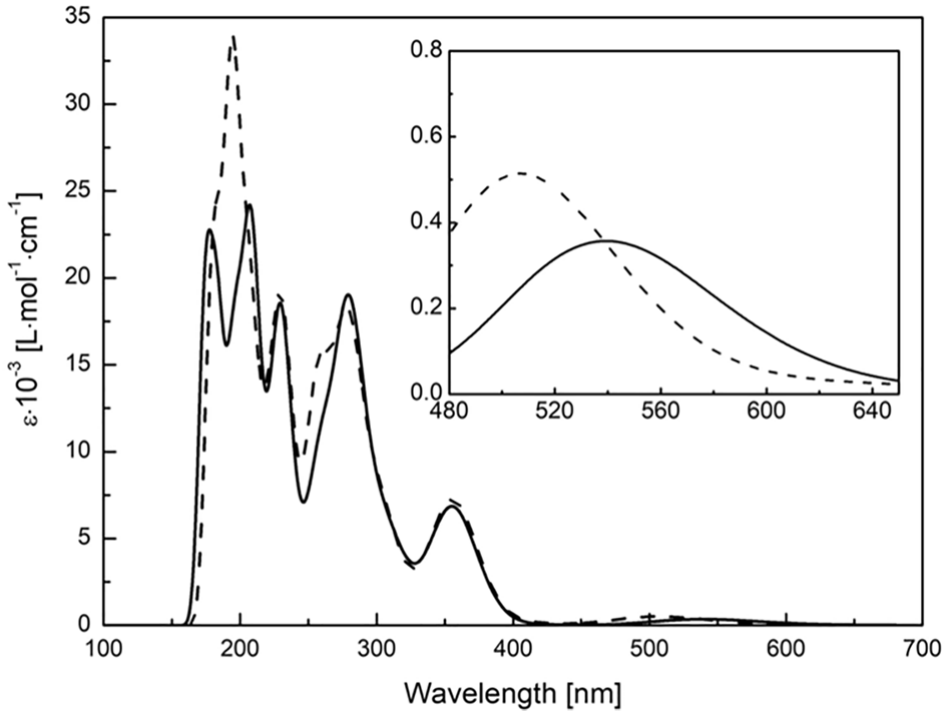
Calculated UV–vis spectra of CuST (solid line) and CuST-Imi (dashed line). All results were obtained using a TD-DFT approximation at the B3LYP/6-311G(d,p) level of theory. Inset: magnification of the spectra in the range of 480–650 nm.

**Figure 5:**
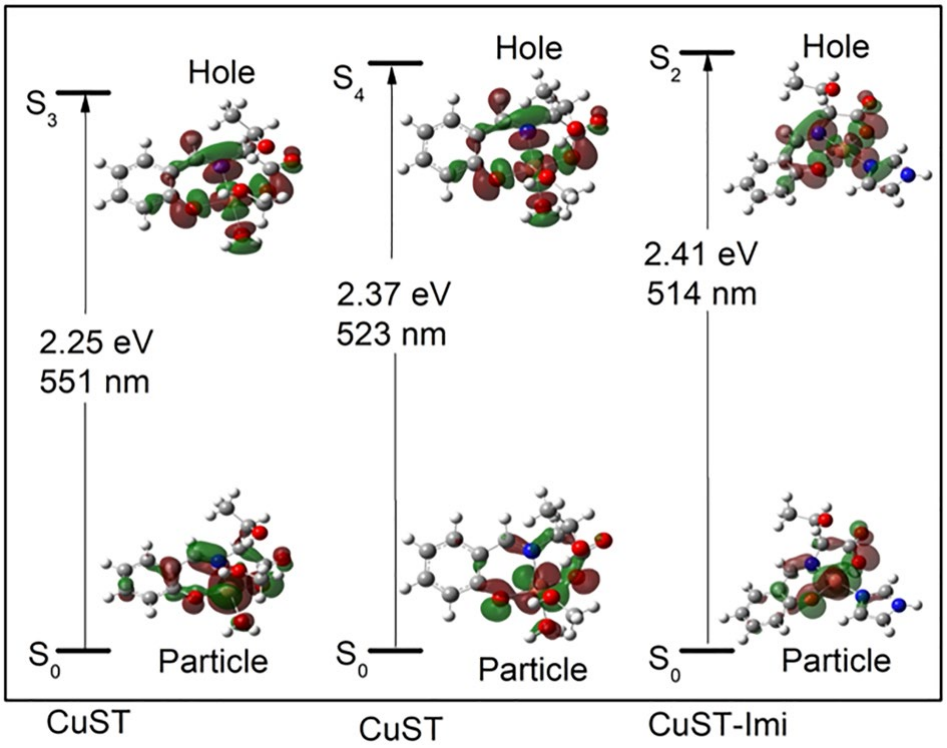
Representation of natural transition orbitals (NTO) of the “particle” and “hole” pairs for the d–d bands of CuST and CuST-Imi. The NTO pairs occupy more than 1.97 of each excited state (isodensity contour 0.03).

The model UV–vis spectra showed the same trend, that is, a blue shift of the maximum (37 nm) accompanied by an increase in intensity (i.e., 356 M^–1^ cm^–1^ for CuST vs. 512 M^–1^ cm^–1^ for CuST-Imi; Fig. 4). To clarify the nature of the d–d transition of the complexes, natural transition orbital (NTO) analysis was performed (Fig. 5). Direct molecular orbital analysis was not informative because the target d–d transitions (S0–S3 and S0–S4) for CuST comprise 16 and 12 electron excitations between the occupied and unoccupied molecular orbitals. Similarly, mixed MO excitation was observed for CuST-Imi. NTO analysis provides a more intuitive picture of the orbitals (mixed or not) involved in hole–particle excitation^37^. This method is very useful for highly complex excitations in MO analysis. The d–d bands for CuST are formed by two excitations, S0–S3 (f = 0.002135761) and S0–S4 (f = 0.003199473), and those for CuST-Imi are formed by an S0–S2 excitation (f = 0.002798322). NTO analysis of the transition density for CuST shows that the most intense electron transfer is intramolecular charge transfer. The distribution of "particle" transition orbitals (occupied) is identical for both studied excitations and is spread over the benzene ring, azomethine, hydroxyl groups, carboxyl groups, and copper d-orbitals, with a small contribution from the coordinated water molecule. The "hole" transition orbital (virtual) is very similar to a "particle,” but with a smaller contribution from the benzene ring, stronger influence of the water molecule, and involvement of only the copper dx^2^-y^2^ orbital. The structure "particle" and "hole" transition orbitals for CuST-Imi are very similar to those of CuST. The only difference arises in the "hole" orbital, where the contribution of the imidazole molecule in CuST-Imi is more significant than that of the water molecule. Along with a change in the geometries of CuST and CuST-Imi, this is the explanation for the observed increase in the intensity of the d–d bands.

### Formation of protein-bound CuST (CuST@lysozyme) in aqueous solution

#### UV–vis spectra of CuST upon binding to lysozyme

**Figure 6:**
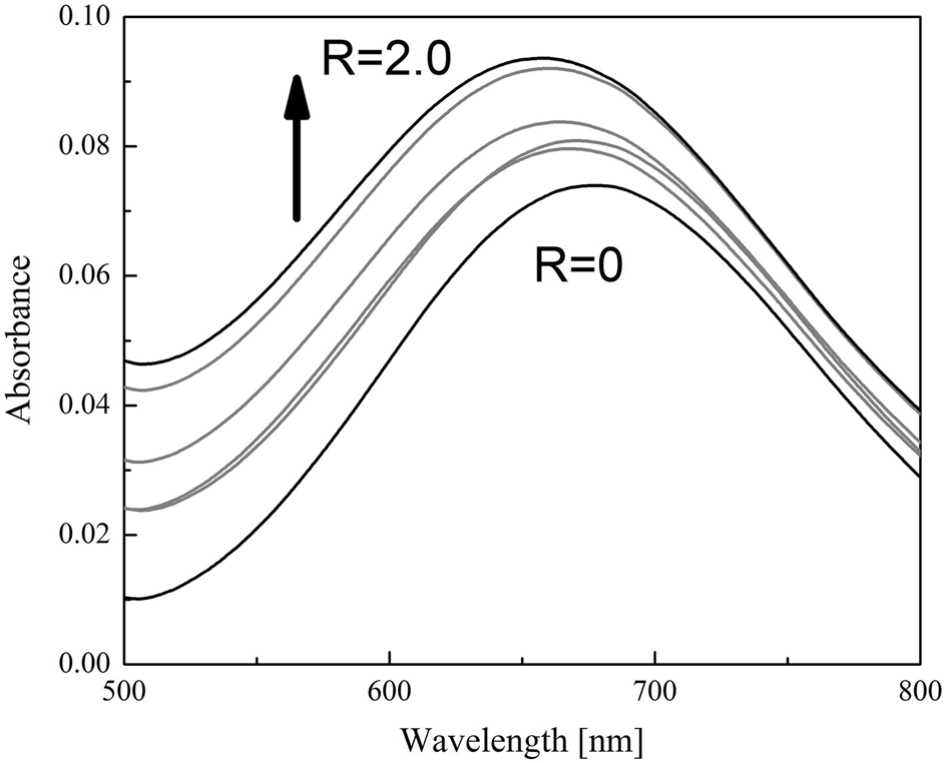
UV–vis spectra of CuST (0.9 mM) in d–d region, under various concentrations of lysozyme (0−1.8 mM) in 0.1 M phosphate buffer (pH 7.0). R: Concentration ratio of lysozyme vs. CuST (C_lysozyme_/C_CuST_). These spectra were measured at every 0.4 steps of R (R = 0, 0.4, 0.8, 1.2, 1.6, 2.0).

To investigate the binding properties of CuST, the UV–vis spectral characteristics of CuST upon the addition of lysozyme were analyzed. The spectra are shown in Fig. 6 (d–d region) and Fig. S2 (full range). In the presence of lysozyme, the d–d transition of CuST shifted from 678 to 658 nm. This blueshift of the d–d transition is consistent with that of obtained by imidazole coordination to CuST (from 678 to 656 nm) and suggests that the imidazole group of the His15 residue binds to the Cu(II) center in the equatorial position. In addition to the blueshift, the intensity of the d–d band increased slightly, indicating that the symmetry around the Cu(II) center was reduced in the presence of lysozyme. In general, imidazole groups are protonated under acidic conditions (pH < 6) to form imidazolium cations. However, they are deprotonated under strongly basic conditions (pH > 14), forming an imidazolate anion. When the pH of the solution is between 6 and 14, the imidazole group is neither protonated nor deprotonated. Therefore, in pH 7.0 solution, imidazole can bind to the Cu(II) center in the neutral state. Although this UV–vis spectral behavior was not sufficiently quantitative to determine the binding constants, the spectral change was qualitatively saturated. In addition, CuST-Imi was obtained through the reaction of CuST with 1 eq. of imidazole in good yield (77.8%). Based on these results, we presume that CuST binds sufficient well with the imidazole group of His15.

### Electrochemical properties of CuST-Imi and CuST@lysozyme

**Figure 7:**
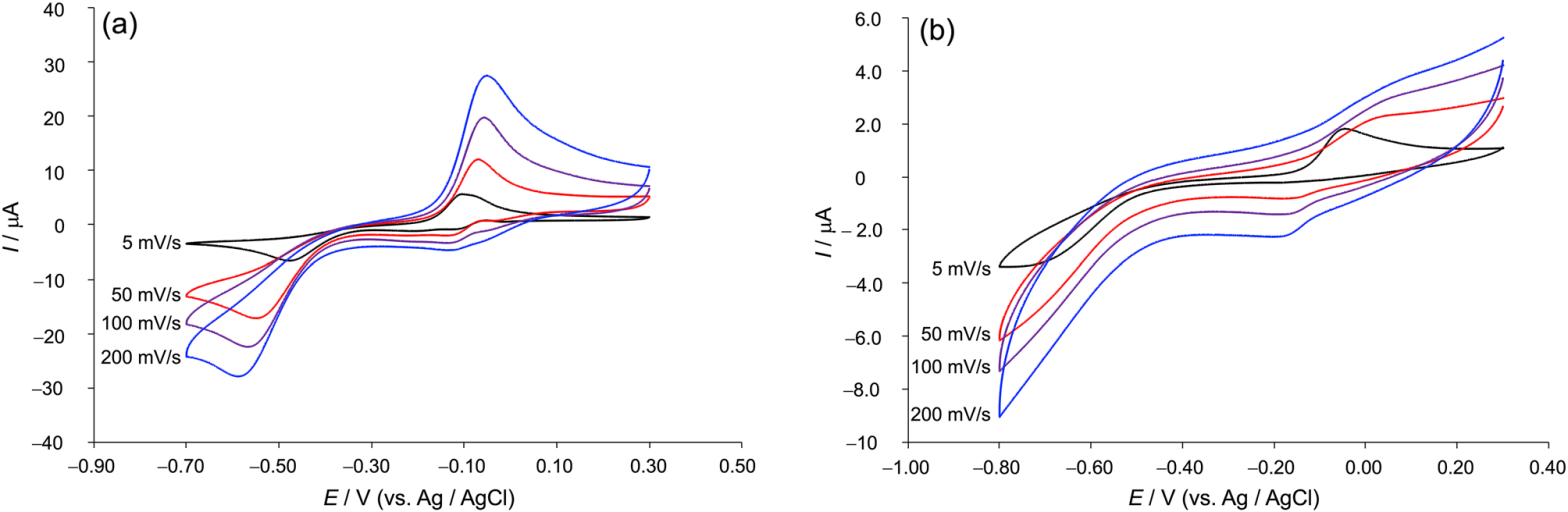
Cyclic voltammograms of (**a**) CuST-Imi (1.0 mM) and (**b**) CuST@lysozyme (CuST 1.0 mM + lysozyme 2.0 mM) at various sweep rates (5−200 mV/s). All voltammograms were measured in 0.1 M phosphate buffer (pH 7.0). Glassy carbon, Pt-wire, and Ag/AgCl electrodes were used as the working, counter, and reference electrodes, respectively.

To understand the electrochemical behavior of CuST-Imi and CuST@lysozyme, cyclic voltammetry (CV) was performed. As a control, a cyclic voltammogram of lysozyme was measured to avoid redox signals in the potential window of the phosphate buffer. The voltammograms of CuST-Imi and CuST@lysozyme are shown in Fig. 7. When the sweep rate was 5 mV/s, CuST-Imi showed oxidative (I_1_) and reductive (I_2_) peaks at − 0.10 and − 0.51 V (vs. Ag/AgCl), respectively. These voltammograms were obtained by starting with negative sweeps from their rest potentials. On the other hand, when anodic waves were not observed in the first scan, positive sweeps were used for the first scan. Therefore, the irreversible redox processes of CuST and CuST@lysozyme were assumed to be Cu(I)/Cu(II). A faster sweep rate resulted in a larger oxidative and reductive wave, resulting in a positive/negative shift in the oxidative/reductive peaks. By contrast, CuST@lysozyme showed oxidative (I_2_) and reductive (I_2_’) peaks at − 0.04 and − 0.72 V, respectively, at a sweep rate of 5 mV/s. In contrast to CuST-Imi, CuST@lysozyme exhibited vague oxidative/reductive waves when the sweep rate was increased. The different voltammogram shapes and electrochemical behaviors derived from various sweep rates imply that CuST binds lysozyme almost quantitatively. The vague oxidative/reductive waves of CuST@lysozyme obtained at a faster sweep rate can be explained by slow electron transfer owing to two phenomena: (1) slower diffusion in solution on account of the large molecular size of CuST@lysozyme; when CuST binds to lysozyme, the molecular size and molecular weight increase significantly compared with those of CuST, and larger molecules diffuse more slowly in solution^38^; and (2) steric inhibition of electron transfer between the Cu(II) center of CuST@lysozyme and the electrode, as the Cu(II) center of CuST@lysozyme is expected to be less accessible to the electrode owing to its large molecular size. These two reasons are consistent with the fact that CuST binds to lysozyme to form a lysozyme–CuST composite. Although these electrochemical results are not sufficient to conclude that CuST binds to lysozyme, we decided that they are sufficient to imply binding. Although the slow electron transfer and low redox potentials of CuST@lysozyme are not suitable for SOD activity, CuST@lysozyme is estimated to show weak SOD activity because O_2_^–^ disproportionation is catalyzed by Lewis-acid-like metal ions.

### Circular dichroism (CD) spectral investigation

Circular dichroism (CD) spectroscopy is an efficient technique for analyzing both the stereochemistry and conformational changes of chiral biopolymers upon interaction with Cu(II) complexes. Therefore, CD spectroscopy was used to monitor the secondary structural changes in lysozyme upon its reaction with CuST.

**Figure 8:**
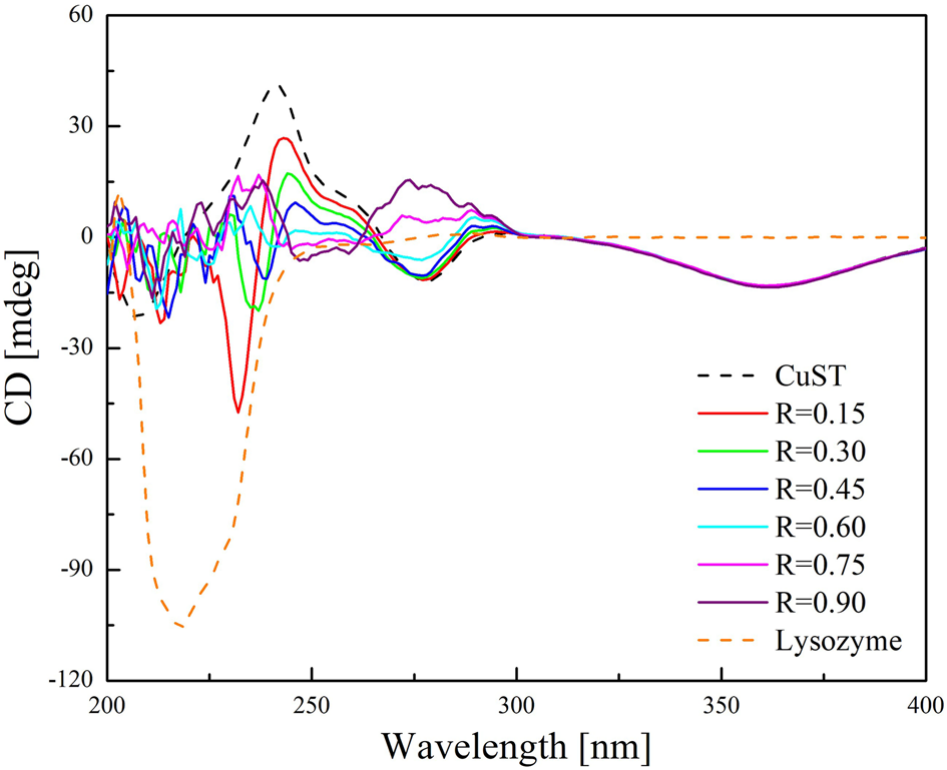
CD spectra of CuST (1.0 × 10^−1^ mM), lysozyme (0.9 × 10^−1^ mM), and their mixture in various ratios (R = C_Lysozyme_/C_CuST_). These CD spectra were measured in phosphate buffer (pH 7.0) with increasing lysozyme concentration (0–0.9 × 10^−1^ mM). A quartz cell (path length: 1.0 cm) was used for the measurements.

The CD spectra of lysozyme, CuST, and their mixtures in various ratios are shown in Fig. 8. Initially, the CD spectrum of lysozyme solution contained a strong negative band at 218 nm and a negative shoulder peak at 232 nm. The observed characteristic signals are consistent with those of lysozymes^39,40^ and proteins with α-helix and β-strand structures. By contrast, CuST showed positive signals at 241 and 258 nm, and negative signals at 278 and 360 nm. These signals were attributed to the chiral carbons of the threonine moiety of CuST^41^. Upon the addition of lysozyme to the CuST solution, the negative band at 278 nm flipped positively. In addition, the CD spectrum is not a simple sum of that of CuST and lysozyme. The CD spectra implied the existence of interactions between CuST and lysozyme. However, the spectral characteristics might also imply that the secondary structure of lysozyme is denatured in the presence of CuST. To investigate the structural changes of lysozyme in the presence of CuST, we performed small-angle X-ray scattering (SAXS) measurements.

### Monitoring of secondary structural changes of lysozyme in the presence of CuST using SAXS

Small-angle X-ray scattering (SAXS) is often used to observe changes in the hierarchical structures of proteins. In the case of lysozyme, which is used in this study, the scattering curves are divided into four q-value ranges, namely, less than 0.2 Å^–1^, ≈ 0.25–0.5 Å^–1^, ≈ 0.5–0.8 Å^–1^, and ≈ 1.1–1.9 Å^–1^, corresponding to (A) the tertiary structure, (B) the interdomain correlation and intradomain structures, (C) the secondary structures, and (D) the closely packed side chains in the hydrophobic cores, respectively^42^.

We prepared solutions containing 3.75, 7.50, and 15.0 mg/mL of lysozyme in phosphate buffer (pH 7.0) and in acetate buffer (pH 4.0). A tenfold concentration of CuST was added to these solutions to obtain their X-ray scattering curves. As shown in Fig. S3, in both buffer solutions, the X-ray scattering curves did not change in the presence of CuST, indicating that no conformational denaturation of lysozyme occurred in the presence of CuST in the aqueous solution.

### Crystal structure of CuST@lysozyme

A single crystal of CuST@lysozyme suitable for crystallographic analysis was obtained by soaking single crystals of lysozyme prepared using the hanging-drop vapor-diffusion method. The overall structure and crystallographic data of CuST@lysozyme are shown in Fig. S4 and Table S2, respectively. The resulting electron density map shows that CuST was adopted on the surface of lysozyme to form a 1:1 composite with 0.7 CuST occupancy. The imidazole group of His15 at the equatorial position of CuST coordinated with the central Cu(II) ion to form a square planar structure.

**Figure 9:**
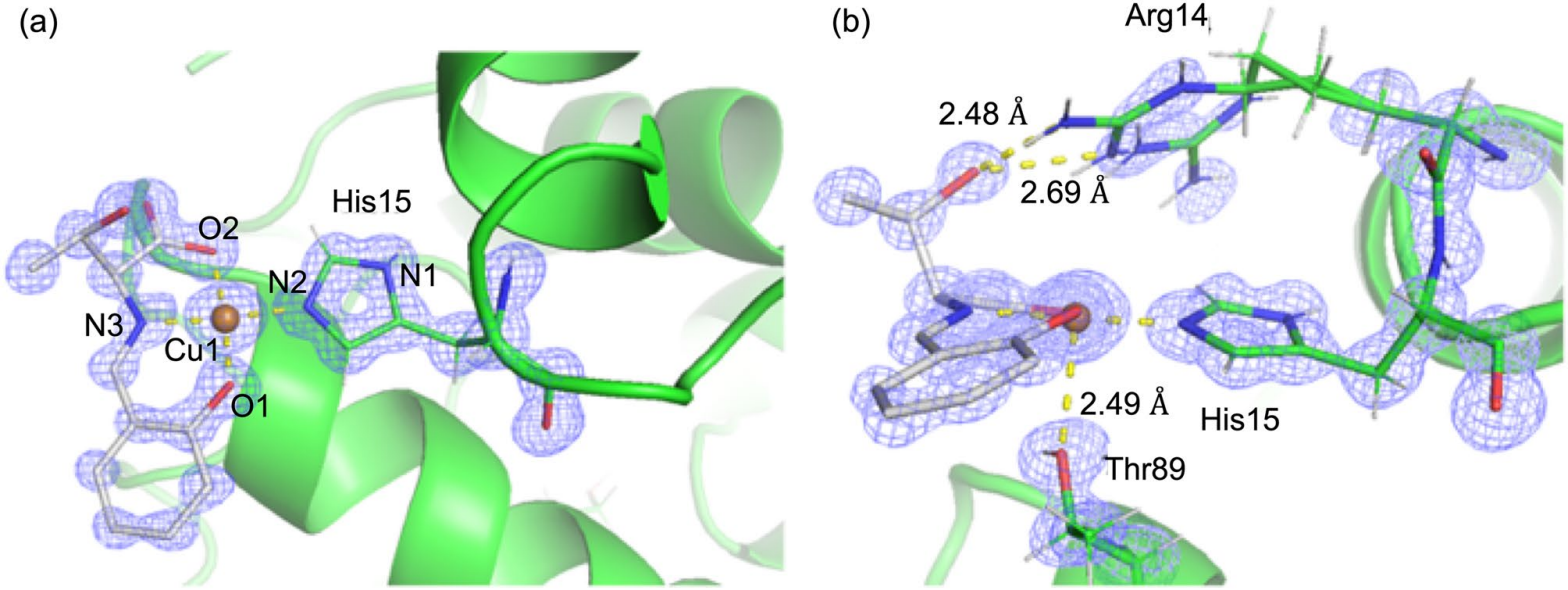
(**a**) Top view and (**b**) side view of crystal structures of CuST@lysozyme around the Cu(II) center. The 2*F*_*o*_* − F*_*c*_ electron-density map is contoured 1.0 σ. *R*_work_/*R*_free_ = 0.1120/0.1291. Selected bond lengths (Å) and angles (°): Cu1–O1 = 1.994, Cu1–O2 = 2.035, Cu1–N2 = 1.979, Cu1–N3 = 1.983, O1–Cu1–N3 = 91.87, N3–Cu1–O2 = 82.62, O1–Cu1–N2 = 94.54, O2–Cu1–N2 = 90.91, O1–Cu1–O2 = 172.48, and N2–Cu1–N3 = 173.56.

The detailed structure around the Cu(II) center of CuST@lysozyme is shown in Fig. 9. In addition to the coordination of the His15 residue to the Cu(II) center of CuST, the side chain of Thr89 was oriented toward the Cu(II) center, forming a weak bond (the distance between Thr86-OH and Cu was 2.49 Å) in the axial position. Furthermore, a comparison of the structures revealed that the side chain of Arg14 moved from its original position to the Cu(II) center upon coordination of the His15 residue and had two conformers with 0.4 and 0.6 occupancies. One of the disordered Arg14 side chains formed hydrogen bonds with the hydroxy group of CuST (2.48 and 2.69 Å). These structural features suggest that the CuST unit of CuST@lysozyme is also fixed by several interactions, including weak coordination and hydrogen bondings, in addition to the coordination of the His15 residue to the Cu(II) center of CuST.

The binding abilities of His15 to metal ions have been reported in some studies that deal with composites composed of lysozyme and metal ions, such as Mn(I)^30^, Ag(I)^31^, Au(I)^32^, Pt(II)^33^, and Rh(III)^43^. The Mn(I)–lysozyme composite was formed by the reaction of lysozyme with [Mn(CO)_3_(H_2_O)_3_]^+^, where the metal center was bound by three carbon monoxide molecules (≈ 1.9 Å). In addition, the imidazole group of His15 (≈ 2.4 Å), and two water molecules (≈ 2.3 Å) weakly interacted to form a distorted octahedral structure^30^. In the Ag(I)–lysozyme composite, the central Ag(I) ion is coordinated by the imidazole group of His15 (≈ 2.1 Å), side chain of Arg14 and Asp87 (≈ 2.5 and ≈ 2.3 Å, respectively), and nitrate anion (≈ 2.0 Å) to form a distorted triangular pyramidal structure^31^. The Au(I)–lysozyme composite was prepared by the reaction of the Au(III) complex with an organic ligand and lysozyme^32^. The Au(III) ion is reduced and loses the organic ligand by hydrolysis upon formation of the composite. The Au(I) ion in the Au(I)–lysozyme composite is bound to the imidazole group of His15 and the side chain of Asn93, forming a linear structure^32^. Pt(II) complexes such as cisplatin ([PtCl_2_(NH_3_)_2_]) also react with lysozyme to give Pt–lysozyme composites^33^. The imidazole group of His15 (2.11 Å), amidato oxygen atom of the main chain (2.03 Å), and two ammonia molecules (2.03 Å) coordinate to the Pt(II) ion to form a distorted trigonal pyramidal structure^33^. Two chloride anions dissociate upon formation of the Pt(II)–lysozyme composite^33^. Rh(III)–lysozyme composites were obtained by the reaction between RhCl_3_ and lysozyme^43^. Side chains of the His15 (2.48 Å) and Arg14 (2.29 Å) bind to the Rh(III) center, forming a bent structure^43^. Other sites of the Rh(III) ions remain vacant ^43^.

Although the metal centers of these metal–lysozyme composites are coordinated by side chains such as His15, the other sites of the metal ions are occupied by inorganic monodentate ligands or remain vacant. Furthermore, in many cases, organic multidentate ligands dissociate via hydrolysis upon the formation of metal–lysozyme composites. It is noteworthy that CuST@lysozyme was formed without the dissociation of the organic multidentate ligand of CuST.

The crystal structures surrounding the Cu(II) centers of CuST-Imi and CuST@lysozyme were compared. Both Cu(II) ions were coordinated by phenolato oxygen (O1), carboxylato oxygen (O2), imidazole nitrogen (N2), and imino nitrogen (N3). The Cu–O1 and Cu–O2 distances of CuST@lysozyme (1.994 and 2.035 Å, respectively) were longer than those of CuST (1.891 and 1.936 Å, respectively), whereas the Cu–N2 and Cu–N3 bond lengths of CuST@lysozyme (1.979 and 1.983 Å, respectively) were slightly longer than those of CuST (1.956 and 1.931 Å, respectively). By contrast, CuST@lysozyme possessed larger N2–Cu–N3 and O1–Cu–O2 bond angles (173.56° and 172.48°, respectively) than CuST-Imi (171.46° and 165.30°, respectively), forming square planar (or square pyramidal) structures with less distortion than in CuST-Imi. These longer bond lengths and smaller distortions around the Cu(II) center of CuST@lysozyme were derived from the weak coordination of the hydroxy group of the Thr86 side chain as well as steric repulsion between the CuST unit and lysozyme. Based on the aforementioned electrochemical results (Fig. 7) and spectroscopic/crystallographic measurements, we confidently determined that CuST binds to lysozyme.

### Evaluation of SOD activity

The crystal structure of CuST@lysozyme contains a guanidinium group belonging to Arg14 near the CuST unit, forming hydrogen bonds with the hydroxy group of the Thr moiety of the ligand. On the other hand, SOD1 also has a guanidinium group belonging to Arg143 over the active site, which attract O_2_^–^ ions to the metal center electrostatically^44^. Therefore, it is expected that CuST@lysozyme will show higher SOD activity than CuST-Imi owing to this electrostatic interaction. The SOD activities of CuST-Imi and CuST@lysozyme were evaluated by measuring their IC_50_ values. To prepare the samples, 2 eq. of lysozyme were added to solutions of CuST-Imi, CuST, and CuCl_2_. Judging from the UV–vis spectra (Fig. 6) and electrochemical characteristics (Fig. 7), CuST@lysozyme formed under these conditions. The IC_50_ values for CuST-Imi, CuST, and CuCl_2_ were 131, 143, and 292 μM, respectively. The SOD activity was significantly higher than that of lysozyme (> > 2000 μM). Both CuST-Imi and CuST showed higher SOD activities than CuCl_2_, indicating that the SOD activity of the CuST unit was retained, even when CuST was bound to lysozyme. These results indicated that lysozyme acquired SOD activity by forming a composite with CuST. Unfortunately, the SOD activity of the CuST unit did not improve upon binding to lysozyme. This is because the hydroxyl group of CuST forms hydrogen bonds with the guanidinium group of Arg14, neutralizing the positive charge. As a result, O_2_^–^ ions cannot form strong electrostatic interactions with the guanidinium group of Arg14, although hydrogen bonds play an important role in fixing the CuST unit to lysozyme.

### Investigation of the coordination behavior of azide ion (N_3_^–^) to the Cu(II) centers of CuST-Imi and CuST@lysozyme

**Figure 10:**
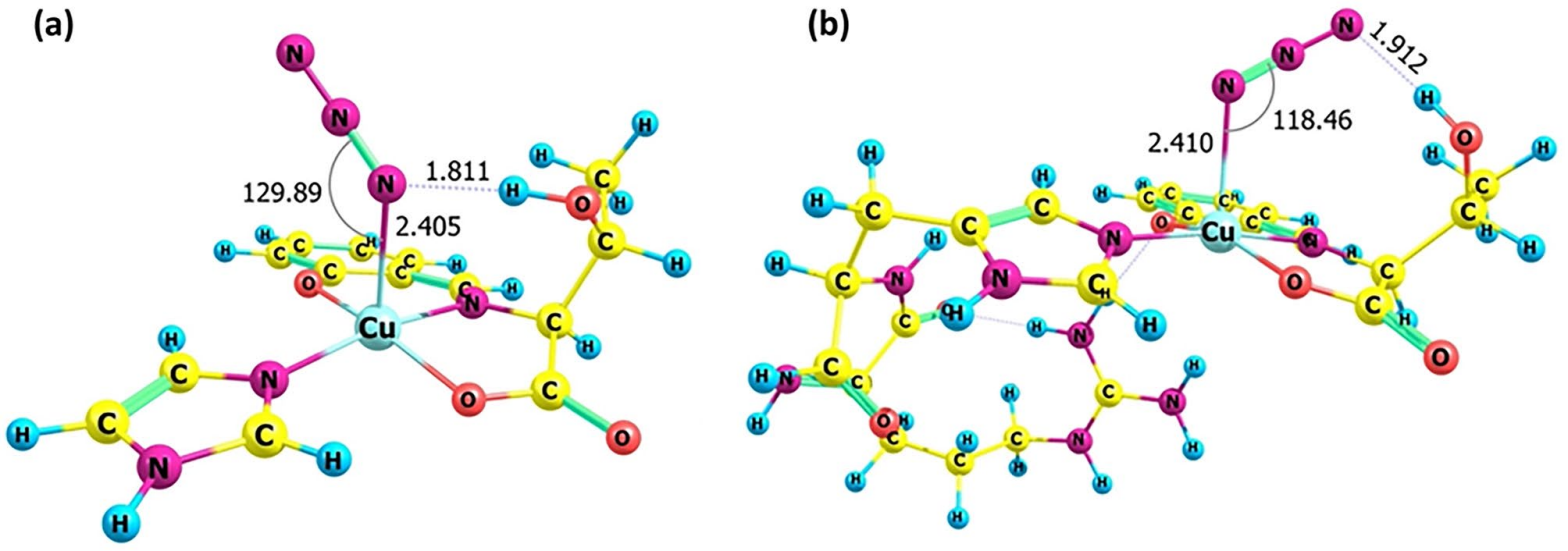
Optimized structures of (**a**) CuST-Imi and (**b**) CuST@lysozyme with N_3_ ion. Selected bond lengths (Å) and angles (°) are shown.

The evaluation of SOD activity revealed that both CuST-Imi and CuST@lysozyme showed higher SOD activities than CuCl_2_. This higher SOD activity prompted us to focus on the disproportionation mechanism of O_2_^–^. The azide ion (N_3_^–^) is widely used as a model anion and inhibitor of O_2_^–^ owing to its size and Lewis basicity, which are similar to those of O_2_^–^^45^. To obtain structural or mechanistic information about the Cu(II) sites of CuST-Imi and CuST@lysozyme upon the disproportionation of O_2_^–^, the coordination properties of the N_3_^–^ ion to the Cu(II) sites were investigated. To clarify the coordination nature of the adducts of CuST-Imi and CuST@lysozyme with the N_3_^–^ ion, quantum-chemical calculations were performed. The optimized structures of the Cu(II) complexes obtained using quantum chemical calculations are shown in Fig. 10. In both cases, the N_3_^–^ ion coordinates to the apical position of the square plane of their Cu(II) centers to adopt a square pyramidal geometry. The formed Cu–N bond is 2.41 Å for both Cu(II) centers. The difference between CuST-Imi and CuST@lysozyme with the N_3_^–^ ion is the nature of the hydrogen bonding between the nitrogen atom of the coordinated N_3_^–^ ion and the hydrogen atom of the hydroxyl group. The same nitrogen atom coordinates with the Cu(II) center and hydrogen-bonds with the CuST-Imi adduct. In CuST@lysozyme, one nitrogen atom forms a coordination bond with the Cu(II) center, and another terminal nitrogen atom forms a hydrogen bond with the hydroxyl group.

### UV–vis spectra of CuST-Imi and CuST@lysozyme in the presence of N_3_^–^

**Figure 11:**
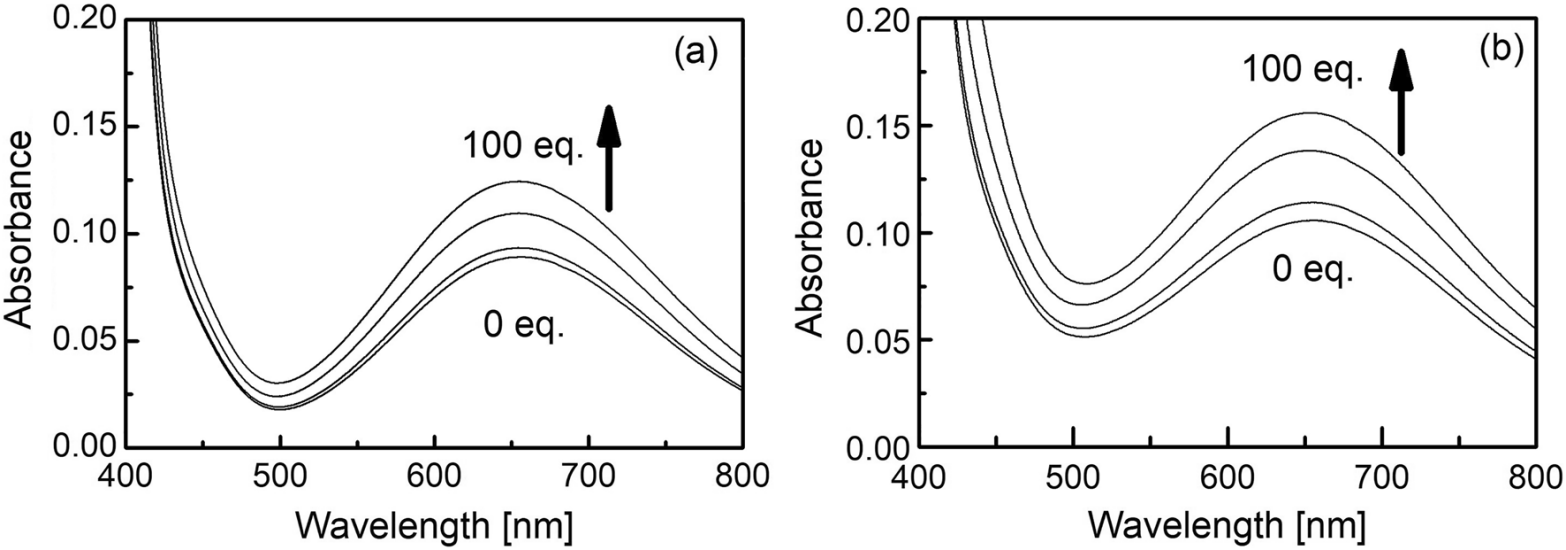
UV–vis spectra of (**a**) CuST-Imi (1.0 mM) and (**b**) CuST@lysozyme (CuST 1.0 mM + lysozyme 2.0 mM) in the presence of 0, 10, 50, and 100 eq. of NaN_3_ in 0.1 M phosphate buffer (pH 7.0).

The UV–vis spectral behavior of CuST-Imi and CuST@lysozyme in the presence of N_3_^–^ ions is shown in Fig. 11. As N_3_^–^ ions were added, the absorption maximum at 657 nm increased for each compound, corresponding to their d–d transitions. These increases in their absorption maxima indicate that the coordination of N_3_^–^ ions to their Cu(II) centers reduces the symmetries in the structures in the vicinity of the central metal ions. The model optical spectra for the adducts of CuST, CuST-Imi, and CuST@lysozyme with N_3_^–^ ions are shown in Fig. S5. The simulated spectra show an increasing intensity of d–d transitions in the region of 400–600 nm upon coordination of N_3_^–^ ions to the Cu(II) complexes. After axial coordination of the N_3_^–^ ion to the Cu(II) centers, a slightly distorted square pyramidal coordination geometry resulted, which was less symmetrical than the initial square planar structure of the Cu(II) complexes.

### Electron paramagnetic resonance (EPR) spectra of CuST-Imi and CuST@lysozyme in the presence of N_3_^–^

**Figure 12:**
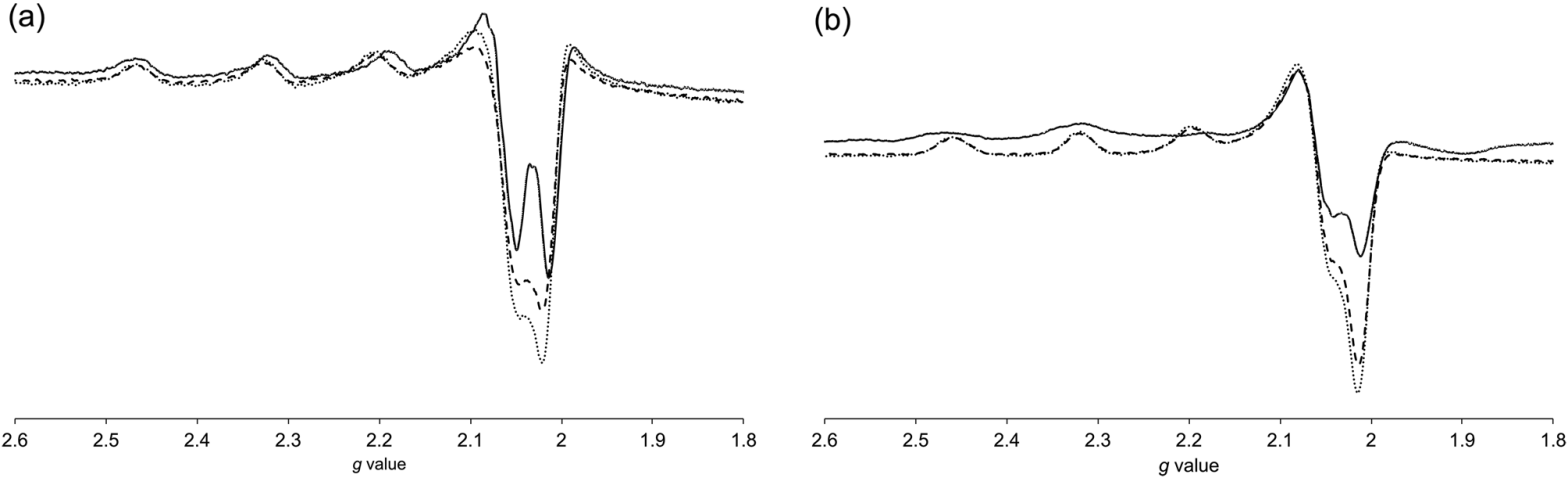
EPR spectra of (**a**) CuST-Imi and (**b**) CuST@lysozyme in the presence of NaN_3_. The bold, dashed, and dotted lines represent the spectra obtained in the absence of NaN_3_, in the presence of 50 eq. of NaN_3_, and in the presence of 100 eq. of NaN_3_, respectively. All samples were prepared as 1 mM solutions of 0.1 M phosphate buffer (pH 7.0) containing 10% ethylene glycol, measured at – 196 ˚C.

**Figure 13:**
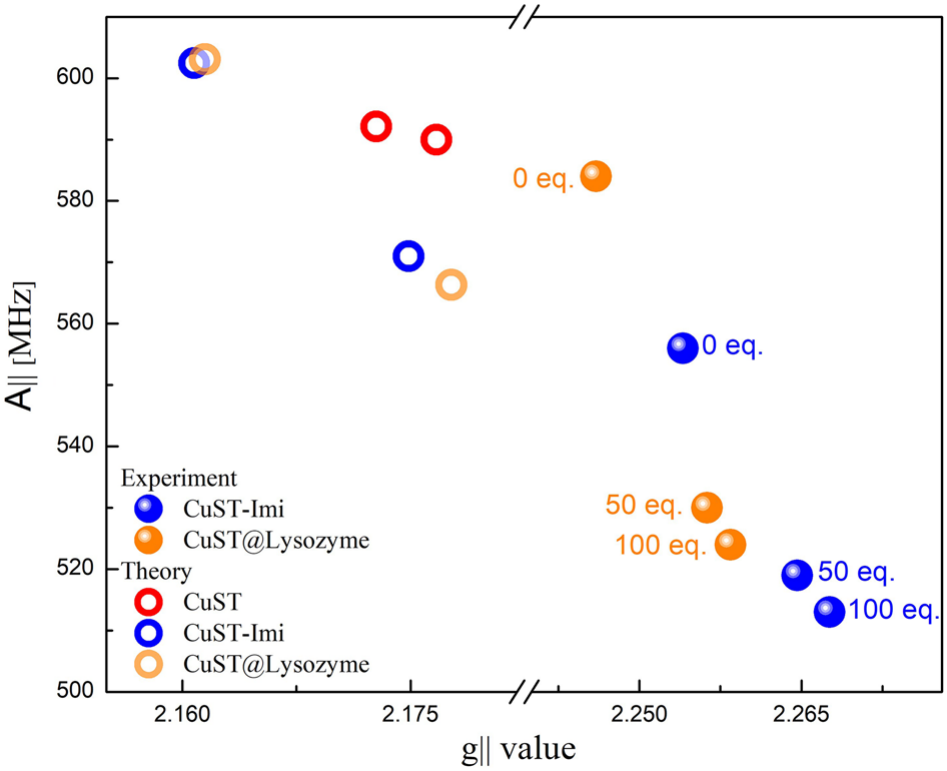
Changes in g_//_ and |A_//_| values of CuST (red circle), CuST-Imi (orange circles) and CuST@lysozyme (blue circles) upon addition of 0, 50, and 100 eq. of NaN_3_. The filled and empty circles denote the experimentally and theoretically obtained values, respectively.

EPR spectroscopy is often used to obtain structural information on Cu(II) complexes owing to its sensitivity to structural changes around the Cu(II) centers. The EPR spectral changes in the presence of N_3_^–^ ions in CuST-Imi and CuST@lysozyme are shown in Fig. 12, comparisons of the measured and simulated EPR spectra are shown in Fig. S6, and the relationships between their g_//_ and |A_//_| values are summarized in Fig. 13 and Table S3. In the absence of the N_3_^–^ ion, CuST-Imi exhibited g_//_ and g_⊥_ values of 2.25 and 2.07, while those for CuST@lysozyme were 2.24 and 2.06, respectively. Both EPR spectra showed larger g_//_ values than g_⊥_ values, indicating that their unpaired electrons were located in their dx^2^-y^2^ orbitals, suggesting that the structures around the Cu(II) center of CuST-Imi and CuST@lysozyme were square planar or square pyramidal^46^. CuST@lysozyme possessed smaller g_//_ and larger |A_//_| values than CuST-Imi (Fig. 13, Table S3). Generally, Cu(II) complexes with distorted square-planar structures show larger g_//_ and smaller |A_//_| values than those of non-distorted square-planar structures ^47^. Based on these tendencies, the smaller g_//_ and larger |A_//_| values of CuST@lysozyme than those of CuST-Imi are consistent with their crystal structures, specifically the smaller distortion in relation to that of CuST-Imi.

In the presence of N_3_^–^ ions, the g_//_ and |A_//_| values of CuST-Imi and CuST@lysozyme gradually shifted, and both compounds exhibited larger g_//_ and smaller |A_//_| values. These shifts are typical of the structural change from square planar to square pyramidal upon axial coordination to Cu(II) complexes with square planar structures^47^. Therefore, these EPR spectral changes indicate that N_3_^–^ ions were axially coordinated to the Cu(II) center of CuST-Imi and CuST@lysozyme. These EPR spectroscopic behaviors are consistent with the crystallographic analysis, as CuST@lysozyme has sufficient space to bind the N_3_^–^ ion on the Cu(II) center.

The theoretically obtained g_//_ and |A_//_| values of CuST-Imi and CuST@lysozyme and their N_3_^–^ adducts were calculated by DFT approximation and are summarized in Fig. 13 and Table S4. In this calculation, the g_//_ and |A_//_| values were evaluated as averages of the g_x_, g_y_, and |A_z_| values. In the CuST, the |A_//_| values slightly decreases since the coordination environment of the Cu(II) ion is not significantly altered because in this reaction, axial coordinated methanol molecule is deposited by N_3_^–^anion. As shown in Fig. 10, the coordination geometries of CuST-Imi and CuST@lysozyme changed from square planar to square pyramidal upon axial coordination of the N_3_^–^ ion. The structural changes are reflected in the g_//_ and |A_//_| values, as follows: DFT modelling revealed that CuST-Imi and CuST@lysozyme showed larger g_//_ values upon coordination of the N_3_^–^ ion, whereas their |A_//_| values 30 (for CuST-Imi) and 37 MHz (for CuST@lysozyme) decreased upon N_3_^–^ coordination. These theoretical results are consistent with the experimentally obtained g_//_ and |A_//_| values upon N_3_^–^ binding.

Based on these findings, O_2_^–^ ions were axially bound to the Cu(II) ion of CuST@lysozyme.

### Theoretical investigation of the O_2_^–^ disproportionation mechanism

**Figure 14:**
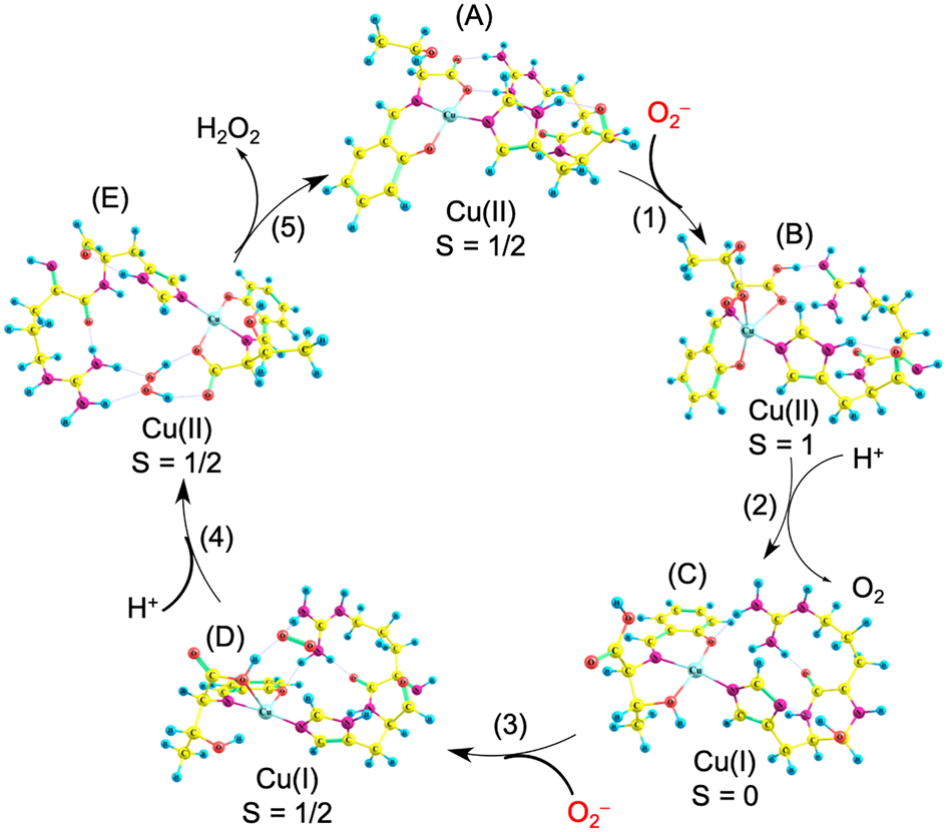
Theoretically proposed O_2_ disproportionation mechanism. A simplified model compound coordinated by an additional ligand with an Arg–His framework (CuST–Arg–His) was employed.

Quantum-chemical calculations were performed to estimate the O_2_^–^ disproportionation mechanism catalyzed by CuST@lysozyme. As a model compound of CuST@lysozyme, a simplified model compound coordinated by an additional ligand with an Arg–His framework (CuST–Arg–His) was employed to save computing resources. Upon estimating the O_2_^–^ disproportionation mechanism, we assumed five states and five steps, as shown in Fig. 14. Five states were assumed: (A) Cu(II) resting state, (B) O_2_^–^-binding Cu(II) state, (C) Cu(I) resting state, (D) O_2_^–^-interacted Cu(I) state, and (E) H_2_O_2_-interacted Cu(II) state. The following steps were also assumed: (1) Cu(II)–O_2_^–^ species-forming step, (2) O_2_^–^-oxidizing step, (3) O_2_^–^-interacted Cu(I) species-forming step, (4) H_2_O_2_-interacted Cu(II) species-forming step, and (5) H_2_O_2_-leaving step.

In the Cu(II) resting state, CuST–Arg–His is in the doublet state because the Cu(II) center has an unpaired electron, and the calculated structure of CuST-Imi shows a slightly square planar geometry. These results were consistent with the crystallographic analysis and EPR spectroscopic properties of CuST-Imi.

In the Cu(II) resting state, O_2_^–^ ions bind to Cu(II) ions to form O_2_^–^-binding Cu(II) species. Owing to the unpaired electrons of Cu(II) and O_2_^–^ ions, singlet or triplet states can be formed. Based on a comparison of their stabilities, the triplet state gives 20.39 kcal/mol lower energy than that of the singlet state (Fig. S7). In this state, O_2_^–^ ions bind to Cu(II) ions to form a distorted square-pyramidal structure. The Cu(II)-binding O_2_^–^ ion formed a hydrogen bond with the hydroxyl group of the ligand. It is worth mentioning that the coordination and hydrogen bonds are formed by the same oxygen atom. A similar hydrogen bond is expected from the optimized structure of N_3_^–^ binding CuST-Imi, as mentioned in Fig. 10.

Under protic conditions, H^+^ interacts with the carboxylate of the O_2_^–^-binding Cu(II) species to form the Cu(I) resting state. In this step, the bound O_2_^–^ ion was oxidized to O_2_ by the Cu(II) ion. In this state, the carboxylate of the ligand was protonated, leaving the Cu(I) ion.

Additional O_2_^–^ ions bind to the resting Cu(I) species to form O_2_^–^-interacted Cu(I) species. In this state, the O_2_^–^ anion does not coordinate to the Cu(I) ion but is fixed by the hydrogen bonds formed between the carboxyl group and guanidinium of the Arg side-chain.

In the O_2_^–^-interacted Cu(I) species, H^+^ ions react with the O_2_^–^ anion to form H_2_O_2_-interacted Cu(II) species. In this state, the H_2_O_2_ molecule also does not bind to the Cu(II) ion but is fixed by four hydrogen bonds with the carboxylate and guanidinium groups. The non-O_2_^–^-coordinating O_2_^– ^reducing mechanism has also been suggested for native CuZnSOD^23^.

In the last step, the H_2_O_2_ molecule interacts with the Cu(II) ion to recover the Cu(II) resting state.

## Conclusion

A novel hybrid protein composed of an SOD-active Cu(II) complex and lysozyme (CuST@lysozyme) was prepared, and its spectroscopic and electrochemical properties were studied. According to the crystallographic analysis, CuST is fixed on the lysozyme by an equatorially coordinated imidazole group of the His15 side-chain, an axially coordinated hydroxyl group of Thr89, and a hydrogen bond between the hydroxyl group of CuST and the guanidinium group of the Arg14 residue (Fig. 9). Both CuST-Imi and CuST@lysozyme showed higher SOD activities than CuCl_2_ and lysozyme. Based on the SOD activity evaluations, lysozyme acquired SOD activity by forming a composite with CuST. The UV–vis and EPR spectra showed that the N_3_^–^ ion was coordinated to the Cu(II) center of CuST@lysozyme (Figs. 11 and 12), suggesting that the O_2_^–^ ion can also coordinate to the Cu(II) center. An O_2_^–^ disproportionation mechanism was proposed based on spectroscopic and quantum chemical simulations. Our study provides valuable insights for the rational design of new SOD therapeutic drug candidates that avoid side reactions owing to decomposition of the SOD mimetic complex. To improve the stability of the hybrid protein in biological fluids such as plasma and cytosol, the following three strategies were undertaken. (1) To suppress ligand dissociation, late-transition-metal complexes were used as binding compounds for lysozyme. (2) To increase the interaction between the complexes and lysozyme, ligands with hydrogen-bonding moieties were used. (3) Since lysozyme contains many basic side chains, the introduction of acidic functional groups to the ligands improved the stability of the metal–lysozyme composites. By improving the stability of the metal–lysozyme composites using these methods, deeper discussions on their therapeutic applications are expected.

## Methods

### Materials and physical measurements

Chemical reagents were purchased from Sigma-Aldrich (St. Louis, MO, USA), FUJIFILM Wako Pure Chemical Corporation (Osaka, Japan), Tokyo Chemical Industry Co. Ltd. (Tokyo, Japan), and Kanto Chemical Co. Inc. (Tokyo, Japan). All reagents were of the highest commercial grade and were used without further purification.

Elemental analyses (C, H, and N) were performed using a Perkin-Elmer 2400 II CHNS/O analyzer at the Tokyo University of Science. IR spectra were recorded on a JASCO FT-IR 4200 spectrophotometer (JASCO, Tokyo, Japan) in the range of 4000–400 cm^−1^ at 298 K. UV–vis spectra were measured using a JASCO V-570 spectrophotometer in the range of 800 − 250 nm at room temperature. The solution concentrations ranged from 0.1 to 1.0 mM, and quartz cells (path length:1.0 cm) were used to contain the samples. The CD spectra were measured using a JASCO J-725 instrument. The solution concentrations were 0.1 mM (CuST) and 0.09 mM (lysozyme), respectively. Quartz cells (path length: 1.0 cm) were used for the measurements. SAXS measurements were carried out at the BL-10C beamline of the 12 keV synchrotron radiation source at the Photon Factory of the High Energy Accelerator Research Organization (KEK, Tsukuba, Japan). The concentrations of lysozyme and CuST were 0.25–1.0 mM and 1.0 mM, respectively. EPR spectra of the complexes were recorded on a Bruker EMX-nano EPR spectrometer (X-band) at 77 K, where 1 mM solutions of CuST-Imi and CuST@lysozyme were measured as samples in quartz tubes. The electrochemical properties of the synthesized complexes were measured using cyclic voltammetry (ALS/DY2323 BI-POTENTIOSTAT) in a 0.1 M phosphate buffer solution (pH 7.0). Glassy carbon, Pt wire, and Ag/AgCl electrodes were used as the working, counter, and reference electrodes, respectively. The CV curves of CuST-Imi (1.0 mM), CuST@lysozyme (CuST 1.0 mM), and lysozyme (2.0 mM) were measured over three cycles under a nitrogen atmosphere at room temperature with a potential sweep rate of 5–200 mVs^−1^.

### Synthesis

#### Preparation of CuST

The Cu(II) complex CuST was synthesized using a published method^34^.

#### Preparation of CuST-Imi

L-threonine (0.119 g, 1 mmol), salicylaldehyde (0.122 g, 1 mmol), and KOH (0.056 g, 1 mmol) were dissolved in 15 mL of methanol and stirred for 1 h at room temperature. A methanol solution (15 mL) of copper (II) acetate monohydrate (0.182 g, 1 mmol) was then added to this solution. After stirring for 15 min, a methanol solution (5 mL) of imidazole (0.068 g, 1 mmol) was added. The reaction mixture was maintained at room temperature for 3–4 days, yielding green, needle-like single crystals suitable for X-ray crystallographic measurements.

Yield: 0.275 g (77.8%). Elemental analysis*.* Calculated for C_14_H_15_N_3_O_4_Cu, C: 47.66%, H: 4.29%, N: 11.91%, Found: C: 47.64%, H: 3.94%, N: 11.79%.

#### Preparation of CuST@lysozyme single-crystal

First, a single crystal of hen egg-white lysozyme (HEWL) was prepared using the hanging-drop vapor-diffusion method. 0.1 M acetic acid-sodium acetate buffer (pH 4.7) containing 2–6% (w/v) NaCl was used as the crystallization solution. Five microliters of lysozyme solution (50 mg/mL) were added to the crystallization solution (5 µL) to form single crystals of lysozyme. The crystals were then transferred to a drop comprising a mixture of 10 µL of crystallization solution and 5 µL of CuST-saturated solution in MeOH; this drop was incubated for a few minutes at 4 °C. The crystals were washed over the original crystallization solution to remove the excess CuST from the crystal surface. The crystals were soaked in a crystallization solution containing 20% ethylene glycol for a few minutes and then flash-cooled in liquid nitrogen.

#### Crystallographic analysis of single-crystal CuST@lysozyme and CuST-Imi

The X-ray intensity data of the CuST-soaked crystals were collected at 100 K using an Eiger X16M (Dectris) detector on BL-17A at the Photon Factory of the High Energy Accelerator Research Organization (KEK, Tsukuba, Japan). The wavelength of synchrotron radiation, the sample-to-detector distance, oscillation range, and exposure time were 0.98 Å, 95 mm, 0.5° (total 180°), and 0.2 s, respectively. The highest resolution was 0.92 Å, processed using XDS^48^. The crystals belonged to the tetragonal space group (*P*4_3_2_1_2), and the structure was solved by molecular replacement with MOLREP^49^ from the CCP4 program package^50^ using a native HEWL model belonging to the same space group (PDB code: 5LYT)^51^ as the search model. Model building and refinement were performed using COOT^52^ and PHENIX^53^ software, respectively. The data collection and refinement statistics are summarized in Table S2. The atomic coordinates and structure factors were deposited in the PDB under accession code 7YRK. Images of the crystal structures were prepared using PyMOL^54^.

A single crystal of CuST-Imi was glued on top of the glass fiber and coated with a thin layer of epoxy resin to measure the diffraction data. The intensity data were collected using a Bruker APEX2 CCD diffractometer with graphite-monochromated Mo-Kα radiation (λ = 0.71073 Å). Data analysis was performed using the SAINT software package. The structures were solved by direct methods using SHELXS-97, expanded by Fourier techniques, and refined using full-matrix least-squares methods based on F2 (SHELXL-97)^55^. An empirical absorption correction was applied using the SADABS program. All non-hydrogen atoms were readily located and refined using anisotropic thermal parameters. All hydrogen atoms were located at geometrically calculated positions and refined using riding models.

#### Evaluation of SOD activities

The SOD activities of the Cu(II) complexes CuST-Imi and CuST@lysozyme were evaluated using the SOD assay kit-WST (Dojindo, Kumamoto, Japan), which is based on the xanthine–xanthine oxidase method, according to the manufacturer’s instructions. The absorbance was measured at 450 nm in a 96-well plate using an iMark Microplate Reader (Bio-Rad Laboratories, Hercules, CA, USA).

#### Computational details

All DFT calculations were performed using the Gaussian 09 program^56^. Full geometric optimization, starting from the available crystallographic structures, was performed for each compound presented in this work. The geometries were optimized using B3LYP^57^ functionals with a split-valence (6-311G(d,p)) basis set^58,59^. All stationary points (SP) were identified as minima (no normal vibrations with imaginary frequencies were detected), and transition states were identified as the first excited states (one imaginary frequency). All open-shell systems were treated with an unrestricted formalism, without symmetry restrictions. To assess the occurrence of spin contamination in the U-DFT calculations, the value obtained for < S2 > was compared to the expected S(S + 1) for the spin state (doublet state). In all the calculations performed, the spin contamination was found to be less than 3% and therefore negligible. The structures of the complexes were visualized using ChemCraft software Ver. 1.6^60^. To model the optical properties, the 120 lowest excitation states were chosen. An increasing number of excitations resulted in bands in the deep-ultraviolet region of the spectrum, which was not the target region of this research. Calculations were performed for the isolated molecules and molecules in the solvent medium (water). A polarizable continuum model was adopted for the latter^61^. The molar absorptivity, ε (L mol^−1^ cm^−1^), was calculated using the GaussSum 3.0 program package^62^. The g-tensor and hyperfine coupling constants (A-tensor) were calculated using the ORCA 5 program package^63^. A hybrid Becke three-parameter functional (B3LYP) was used in combination with the Pople basis set (6-311G(d,p)). The A-tensor was obtained as the sum of three contributions: the isotropic Fermi contact (A^*FC*^), anisotropic dipolar (${\mathrm{A}}_{x, y, z}^{D}$), and spin–orbit coupling term ${(\mathrm{A}}_{x,y,z}^{SO})$. This approximation reproduces the target parameters^64,65^.

## Supplementary Information

Supplementary Information.

## Data Availability

The crystallographic data and diffraction intensities (CIF) reported in this paper have been deposited at the Cambridge Crystallographic Data Centre (CCDC) under reference number (2194652). The atomic coordinates and structure factors for CuST@lysozyme were deposited in the Protein Data Bank (PDB) with accession code 7YRK. All data are available in the main text or supplementary information, and/or from the corresponding author upon request.
