# Supplementary material for: A novel hybrid protein composed of superoxide-dismutase-active Cu(II) complex and lysozyme
Source: Sci Rep. 2023 Apr 27;13:6892. doi: 10.1038/s41598-023-33926-1 (PMC10140267; doi:10.1038/s41598-023-33926-1)
Supplement: Supplementary file 1 — Supplementary Information. [file 41598_2023_33926_MOESM1_ESM.docx]

**Supplementary Information for**

**A Novel Hybrid Protein Composed of Superoxide-Dismutase-Active Cu(II) Complex and Lysozyme**

**Contents**

Table S1. Crystallographic data collection and refinement statistics for CuST-Imi.

Figure S1. FT-IR spectra of CuST and CuST-Imi.

Figure S2. Full-range UV–vis spectral changes for CuST in the presence of 0−2.0 eq. of lysozyme.

Figure S3. Wide-angle X-ray scattering curves of lysozyme in the presence of CuST.

Figure S4. Overall structure of CuST@lysozyme.

Table S2. Crystallographic data collection and refinement statistics for CuST@lysozyme.

Figure S5. Calculated UV–vis spectra of CuST, CuST-Imi, CuST-Imi@lysozyme, and the corresponding adducts with N_3_^−^ ions.

Figure S6. Comparison of measured and simulated EPR spectra of CuST-Imi and CuST@lysozyme in the presence of NaN_3_.

Table S3. Experimentally obtained g and |A_//_| values of CuST and CuST@lysozyme and their N_3_^−^ adducts in varying amounts.

Table S4. Theoretically obtained g and |A| values computed for CuST, CuST@lysozyme, and their N_3_^−^ adducts.

Figure S7. Structural comparison of CuST–Arg–His in O_2_^−^-binding Cu(II) state.

Table S1. Crystallographic data collection and refinement statistics for CuST-Imi.

| Crystallographic data (CCDC 2194652) | |
| --- | --- |
| Empirical formula | C_14_ H_15_ Cu N_3_ O_4_ |
| Crystal system | orthorhombic |
| Space group | *P* 2_1_ 2_1_ 2_1_ |
| *Z* | 4 |
| *a* / Å | 5.28300(10) |
| *b* / Å | 11.6171(3) |
| *c* / Å | 23.9929(7) |
| *V* / Å^3^ | 1472.52(6) |
| *ρ* calc (g/cm^3^) | 1.592 |
| *μ* (mm^-1^) | 1.505 |
| F ( 0 0 0 ) | 724 |
| Goodness of fit | 1.029 |
| *R_1_* [I>2σ(I)] | 0.0287 |
| *wR_2_* | 0.0667 |


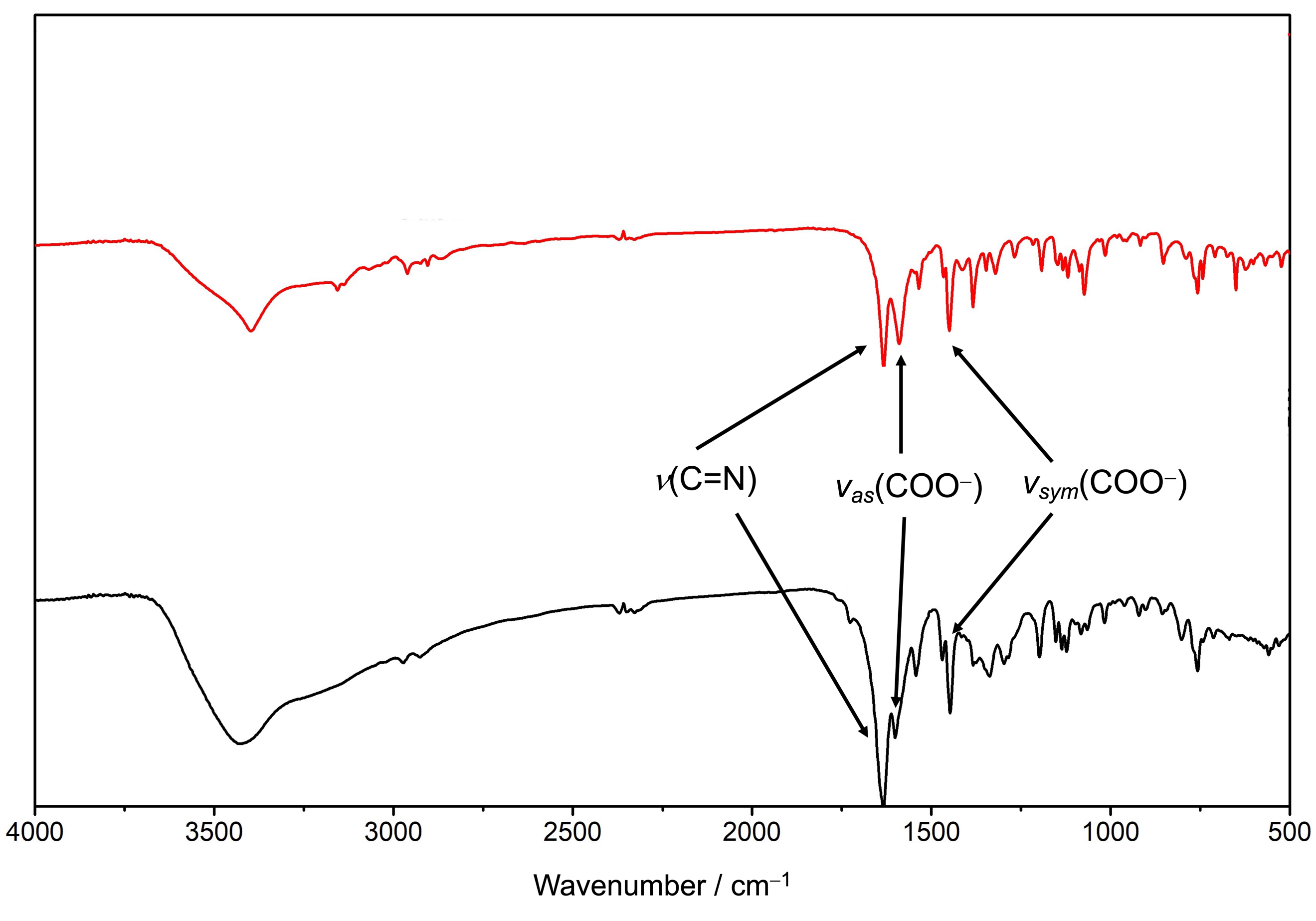
Figure S1. FT-IR spectra of CuST-Imi (red) and CuST (black).

Figure S2. Full-range UV–vis spectra of CuST (50 μM) in the presence of 0−2.0 eq. of lysozyme in 0.1 M phosphate buffer (pH 7.0).
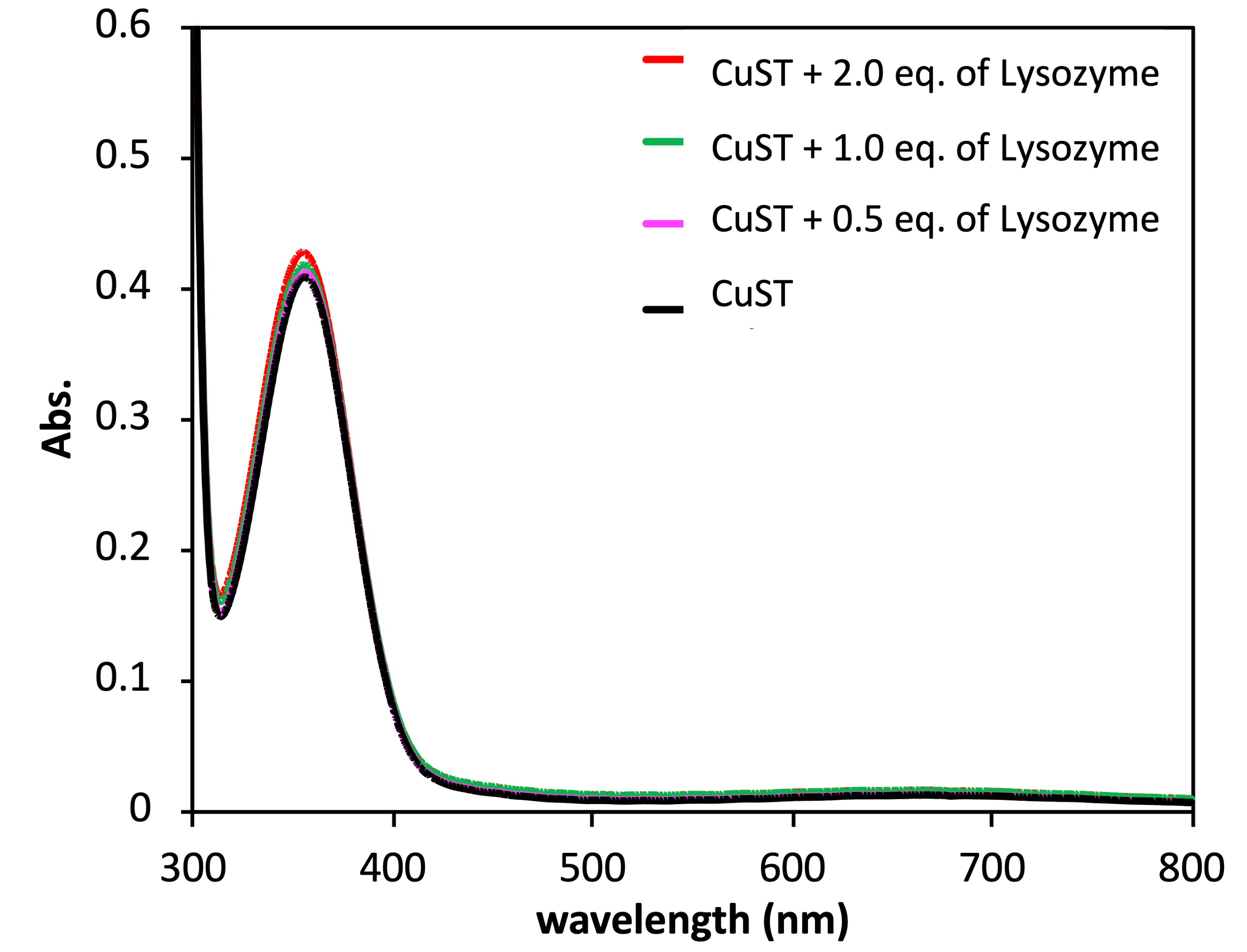


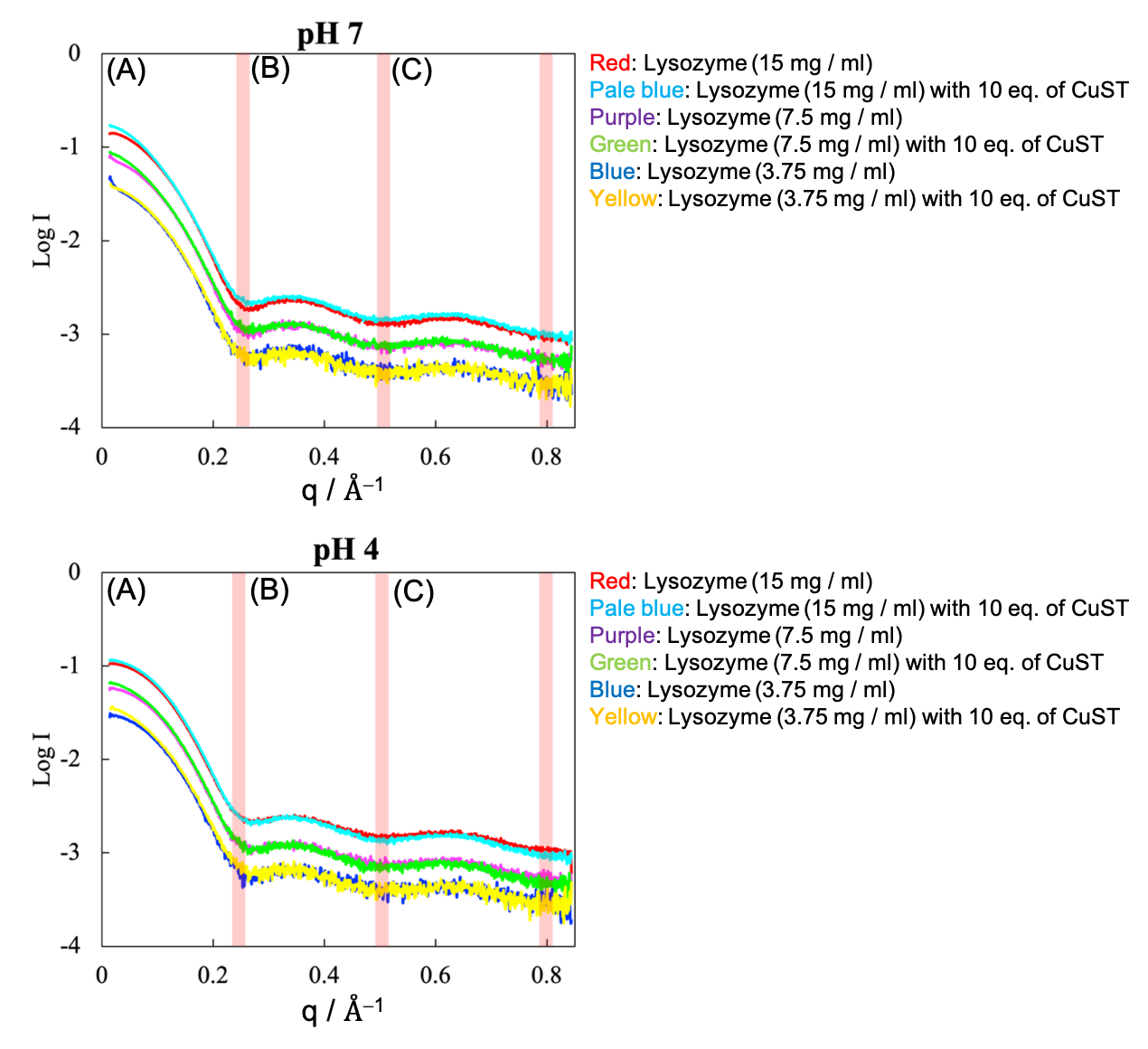


Figure S3. Wide-angle X-ray scattering curves of lysozyme in the presence of 10 eq. of CuST. The lysozyme concentrations were 15.0, 7.50, and 3.75 mg/mL in phosphate buffer (pH 7.0) and acetic acid buffer (pH 4.0). (A), (B), and (C) correspond to the tertiary structure, interdomain correlation, intradomain structures, and secondary structures, respectively.


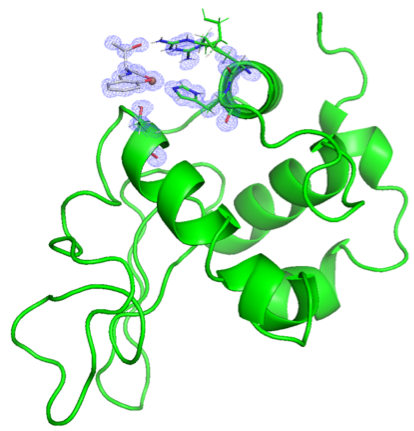


Figure S4. Overall structure of CuST@lysozyme. The different Fourier maps were contoured at 3.0 σ.

Table S2. Crystallographic data collection and refinement statistics of CuST@lysozyme.

| X-ray intensity data (PDB 7YRK) | |
| --- | --- |
| Beamline | PF BL-17A |
| Resolution range | 33.63-0.924 (0.9567-0.924) |
| Space group | *P*4_3_2_1_2 |
| Cell parameters (Å, °) | *a* = *b* = 78.671, *c* = 37.203, α = β = γ = 90 |
| No. of molecules in asymmetric unit | 1 |
| *R*_merge_ (%) | 0.084 (0.497) |
| I/σ | 17.0 (2.0) |
| Multiplicity | 11.6 (3.9) |
| Completeness (%) | 98.09 (82.95) |
| Structure refinement | |
| Resolution range (Å) | 35.18-0.92 |
| *R*_work_ | 0.1120 |
| *R*_free_ | 0.1291 |
| Ramachandran favored (%) | 99.21 |
| Ramachandran outlier (%) | 0.00 |
| RMS bond length (Å) | 0.013 |
| RMS bond angle (º) | 1.16 |


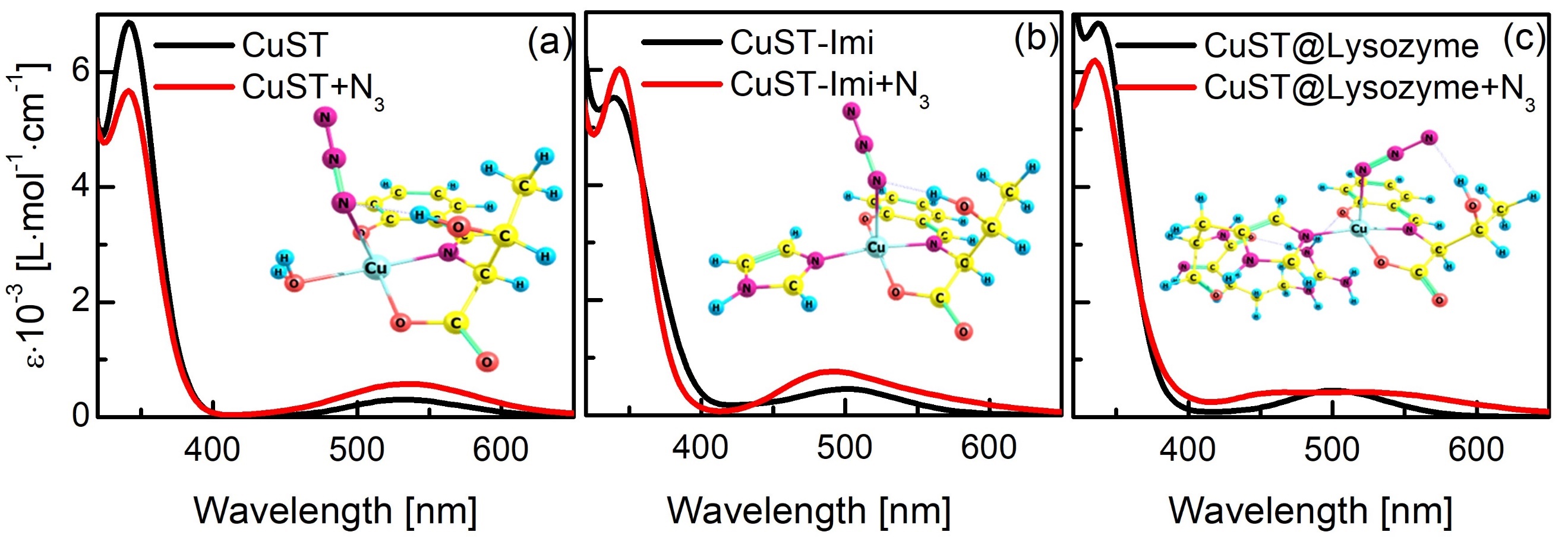


Figure S5. Calculated UV–vis spectra of (a) CuST (black solid line), (b) CuST-Imi (black solid line), (c) CuST-Imi@lysozyme (black solid line), and the corresponding adducts with N_3_^−^ ion (red lines). All results were obtained using TD-DFT approximation at the B3LYP/6-311G(d,p) level of theory.


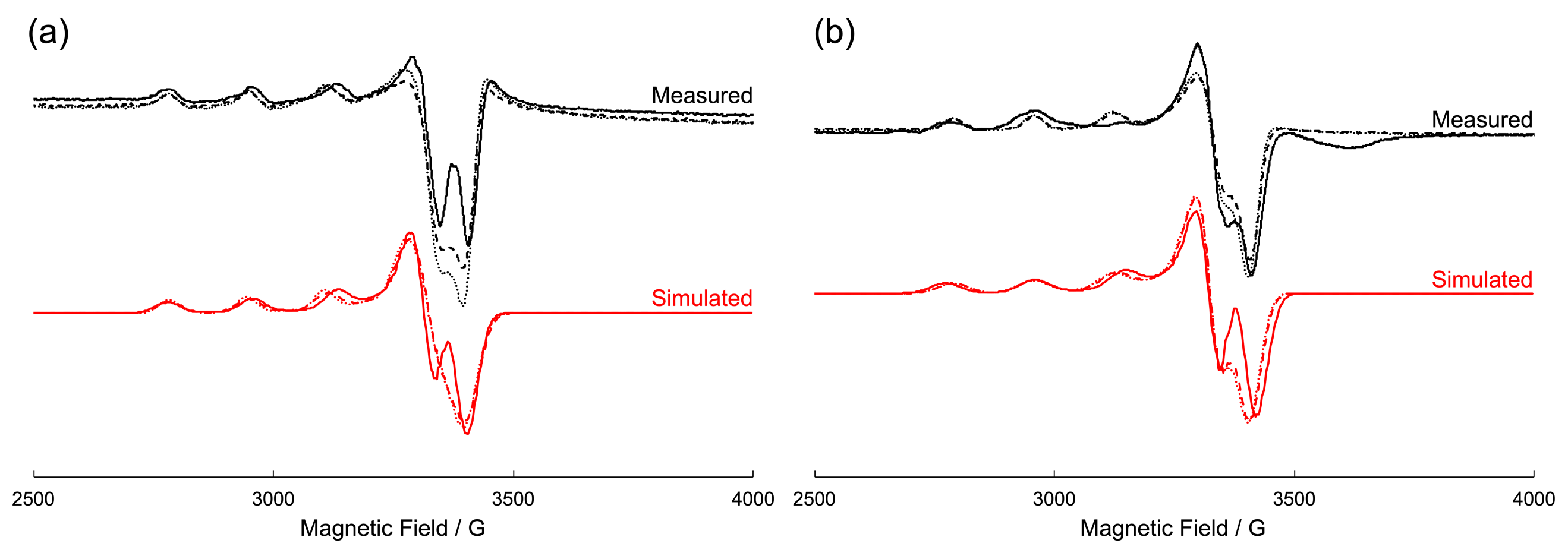


Figure S6. Comparisons of the measured (black lines) and simulated (red lines) EPR spectra of (a) CuST-Imi and (b) CuST@lysozyme in the presence of NaN_3_. The bold, dashed, and dotted lines are the EPR spectra obtained in the absence of NaN_3_, in the presence of 50 eq. of NaN_3_, and in the presence of 100 equiv. of NaN_3_, respectively. All samples were prepared as 1 mM solutions of 0.1 M phosphate buffer (pH 7.0) containing 10% ethylene glycol, measured at −196 °C.

Table S3. Experimentally obtained g and |A_//_| values of CuST and CuST@lysozyme and their N_3_^−^ adducts in varying amounts.

| g values | | | | |
| --- | --- | --- | --- | --- |
| Compound |  | 0 eq. N_3_^−^ | 50 eq. N_3_^−^ | 100 eq. N_3_^−^ |
| CuST-Imi | g_//_ | 2.254 | 2.264 | 2.268 |
|  | g_⊥_ | 2.070 | 2.062 | 2.061 |
| CuST@lysozyme | g_//_ | 2.246 | 2.256 | 2.258 |
|  | g_⊥_ | 2.061 | 2.127 | 2.127 |
| \|A_//_\| / MHz | | | | |
| Compound |  | 0 eq. N_3_^−^ | 50 eq. N_3_^−^ | 100 eq. N_3_^−^ |
| CuST-Imi |  | 566 | 519 | 513 |
| CuST@lysozyme |  | 584 | 530 | 524 |

Table S4. Theoretically obtained g and |A| values computed for CuST, CuST@lysozyme, and their N_3_^−^ adducts.

| Compound |  | g_x_ | g_y_ | g_z_ |
| --- | --- | --- | --- | --- |
| CuST-Imi |  | 2.044 | 2.055 | 2.161 |
| CuST-Imi-N_3_^−^ |  | 2.057 | 2.059 | 2.175 |
| CuST@lysozyme |  | 2.045 | 2.054 | 2.161 |
| CuST@lysozyme-N_3_^−^ |  | 2.059 | 2.060 | 2.178 |
| Compound | Atom | \|A_x_\| | \|A_y_\| | \|A_z_\| |
| CuST-Imi | Cu | 4.70 | 23.70 | 602.46 |
| CuST-Imi-N_3_^−^ | Cu | 21.19 | 40.54 | 570.99 |
| CuST@lysozyme | Cu | 4.10 | 18.98 | 603.08 |
| CuST@lysozyme-N_3_^−^ | Cu | 53.92 | 12.91 | 566.33 |


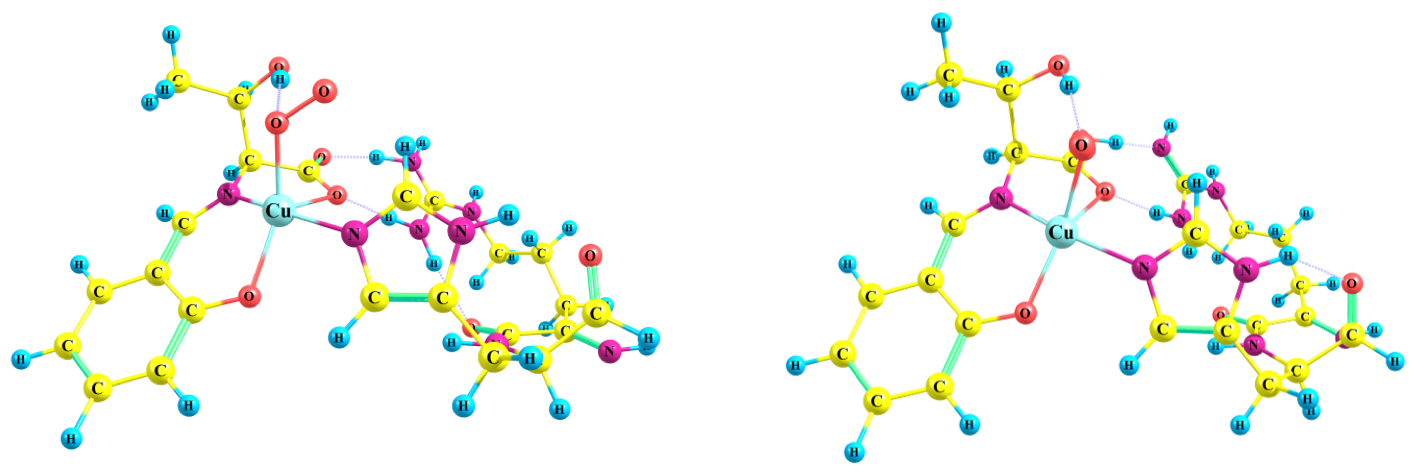


Figure S7. Theoretically estimated structures of CuST–Arg–His in the O_2_^−^-binding Cu(II) state. The triplet (right) state has 20.39 kcal/mol lower energy than that of the singlet state (left).
